# Supplementary material for: Piezo-catalysis for nondestructive tooth whitening
Source: Nat Commun. 2020 Mar 12;11:1328. doi: 10.1038/s41467-020-15015-3 (PMC7067860; doi:10.1038/s41467-020-15015-3)
Supplement: Supplementary file 1 — Supplementary Information [file 41467_2020_15015_MOESM1_ESM.pdf]

Supplementary information for

Piezo-catalysis for nondestructive tooth whitening

Wang et al.

Supplementary Figures 1-17

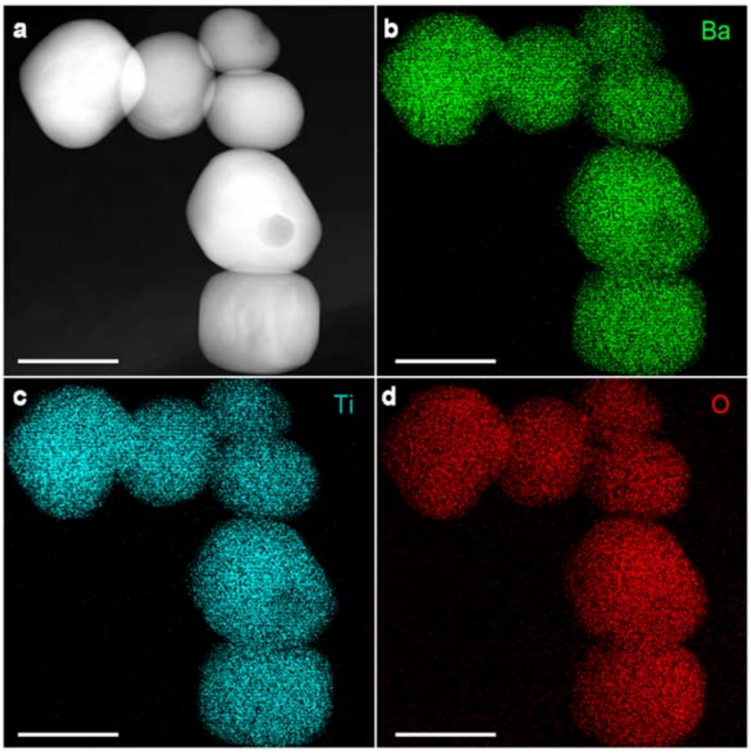

Supplementary Fig. 1 a STEM and **b-d** corresponding EDX element mapping of Ba (greed), Ti (blue), and O (red) in $BaTiO_3$ nanoparticles. Scale bars are 200 nm.

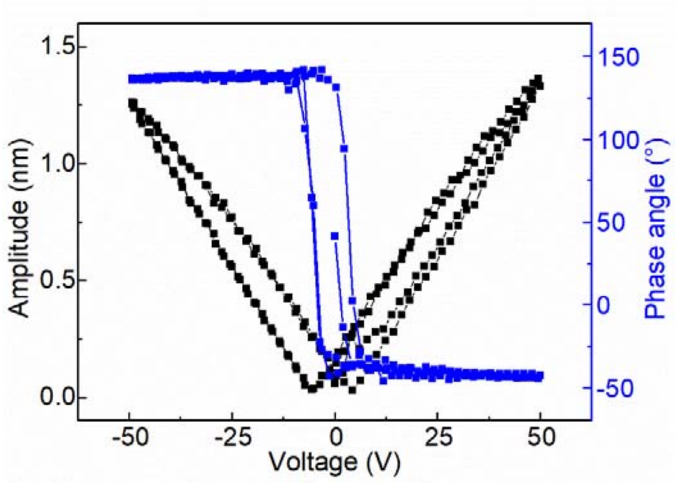

Supplementary Fig. 2 The local piezoelectric hysteresis loop at “on” state.

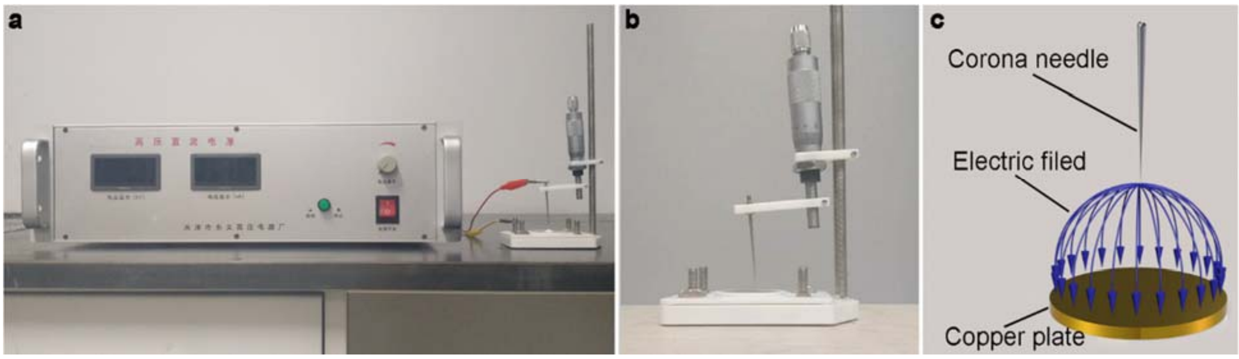

Supplementary Fig. 3. a The setup we used to pole the BTO nanoparticles, **b** the lab-made corona-poling device. The device consists of a sharp needle and a smooth copper plate. When a high voltage was applied on the needle **c**, the actual voltage near the sharp point is much higher due to the tip discharge effect, and in turn the electric field distribution is like an umbrella.

This setup primarily consists of two parts, one is the sharp metallic needle (corona needle), and the other is a plate made by Cu with a smooth surface and large-diameter (Supplementary Fig. 3b). When the high voltage is applied on the needle, since the point discharge, the voltage around the sharp point would be extremely higher than elsewhere, and it can ionize a neutral fluid(air) to form a plasma that consists of electrons and positive ions (Supplementary Fig. 3c). These charges will be separated by the electric filed and impact with other atoms, producing more charges constantly. The electrons are attracted to the needle when it is chosen as the positive electrode, accordingly, and the positive charge is repelled to the upper surface of materials laid on the copper plate[1]. Then, we got the poled nanoparticles of BTO without any mechanical approach.

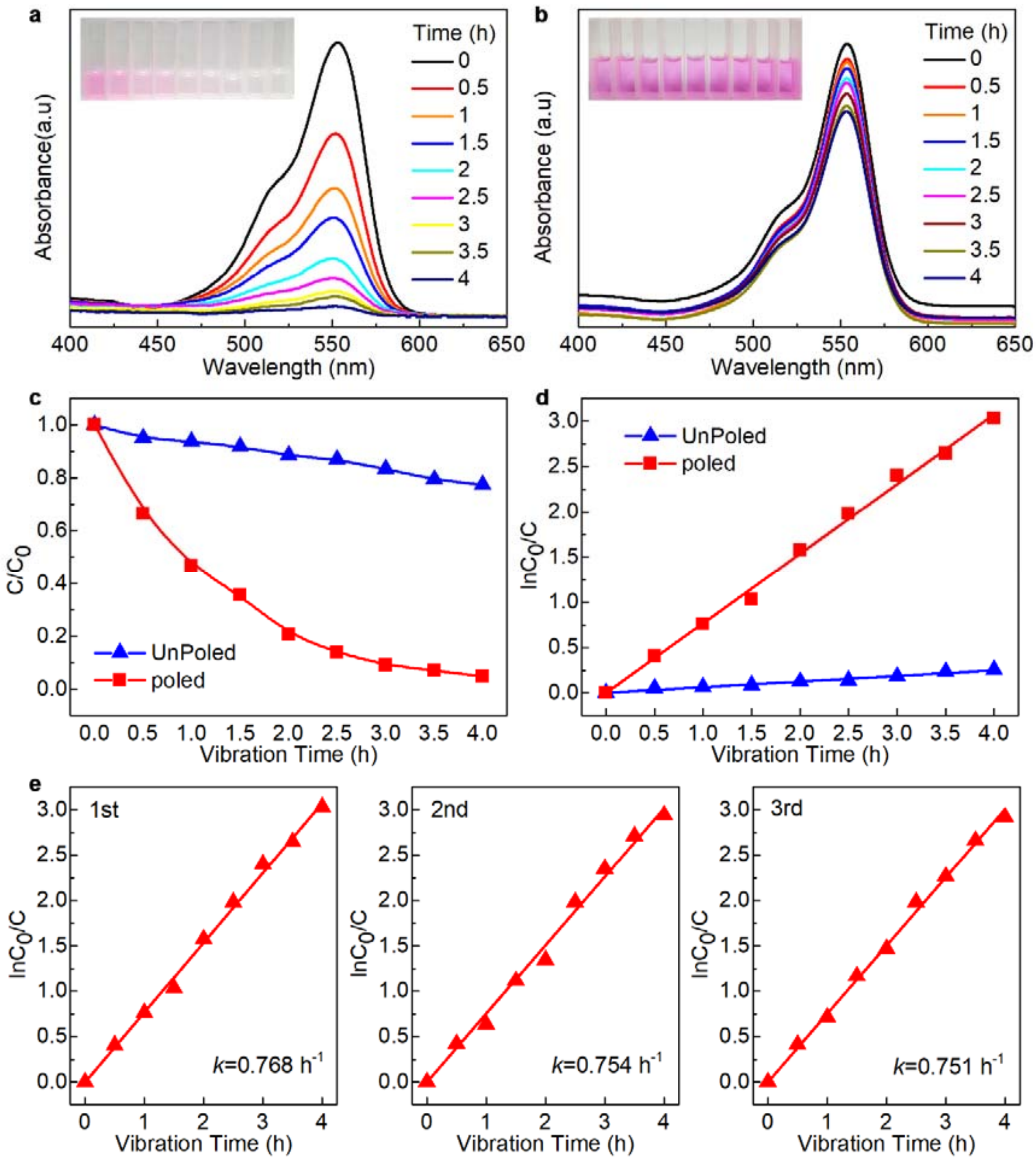

Supplementary Fig. 4 Degradation properties of piezo-catalysis. UV-Vis absorption spectra of RhB solutions at various vibration time for the **a** poled and **b** unpoled BTO nanoparticles. The inset in each Figure is a series of photographs of piezo-catalyzed RhB dye solution progressing in time from left to right. Piezo-catalytic degradation efficiency performance of the poled and unpoled BTO nanoparticles in **c** direct concentration ratio C/C_0 and **d** logarithmic relationship of $\ln(C_0/C)$ by fitting with a linear function. **e** Recycling ability of poled BTO for degrading RhB under an ultrasonic vibration.

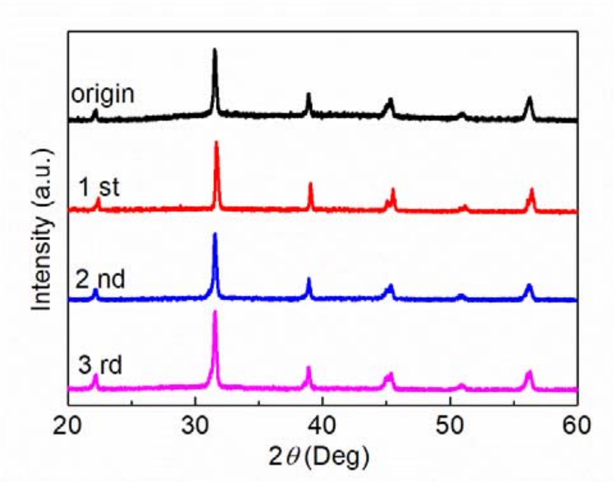

Supplementary Fig. 5 XRD pattern of the BTO nanoparticles before and after several times catalyst test. No additional peaks were observed after the piezo-catalysis process.

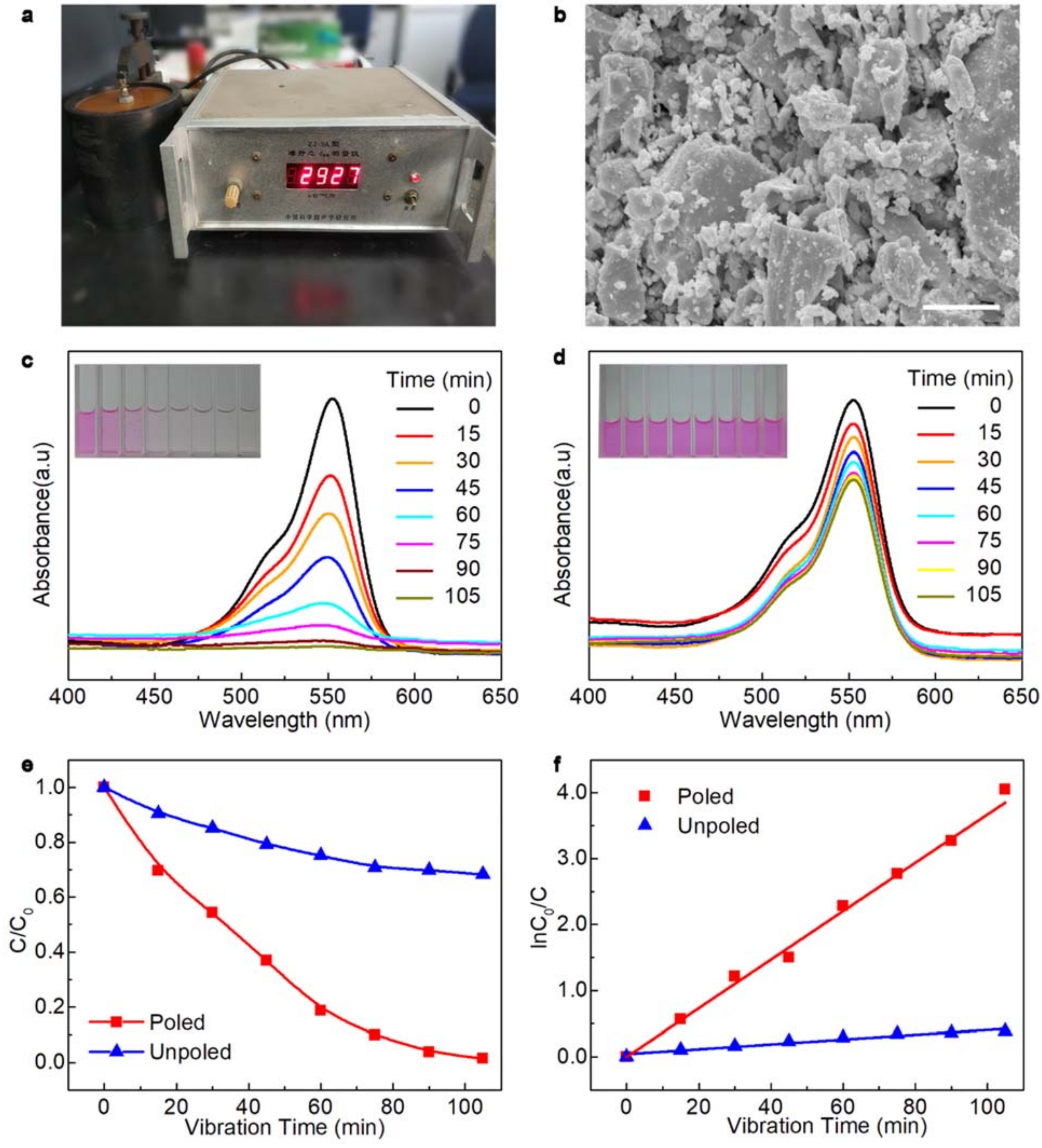

Supplementary Fig. 6 a The Photograph of piezoelectricity measurement, and it shows that d_{33} of [001]-oriented PMN-PT is as high as ~2930 pC N^{-1} . After ball milled for 24 hours, the PMN-PT single crystal has been smashed into little pieces, the size can be seen from **b** SEM image, which range from hundreds of nano-meter to several micro-meter. UV-Vis absorption spectra of RhB solutions at various vibration time for the **c** poled and **d** unpoled PMN-PT single crystal stones. The inset in each figure is a series of

photograph of piezo-catalyzed RhB dye solution, which reveals that the RhB solution became clear with poled PMN-PT single crystal stones after vibration for 105 min, while it shows negligible color change with unpoled PMN-PT ones under the identical condition. Piezo-catalytic degradation efficiency performance of the poled and unpoled PMN-PT single crystal stones in **e** direct concentration ratio C/C_0 and **f** logarithmic relationship of $\ln(C_0/C)$ by fitting with a linear function. Scale bar in b is 5 μm.

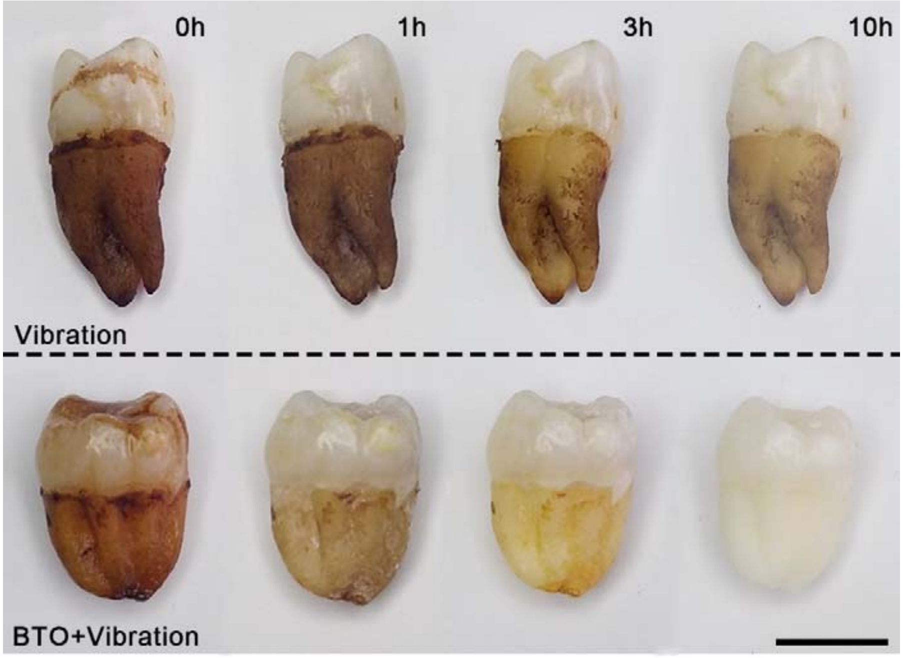

Supplementary Fig. 7. Photographs of teeth stained by black tea under treatment of vibration in (top) pure deionized water and (bottom) turbid liquid of BTO nanoparticles for 0, 1, 3 and 10 hours, respectively. These photographs are successive images of the same tooth. Scale bar is 1 cm.

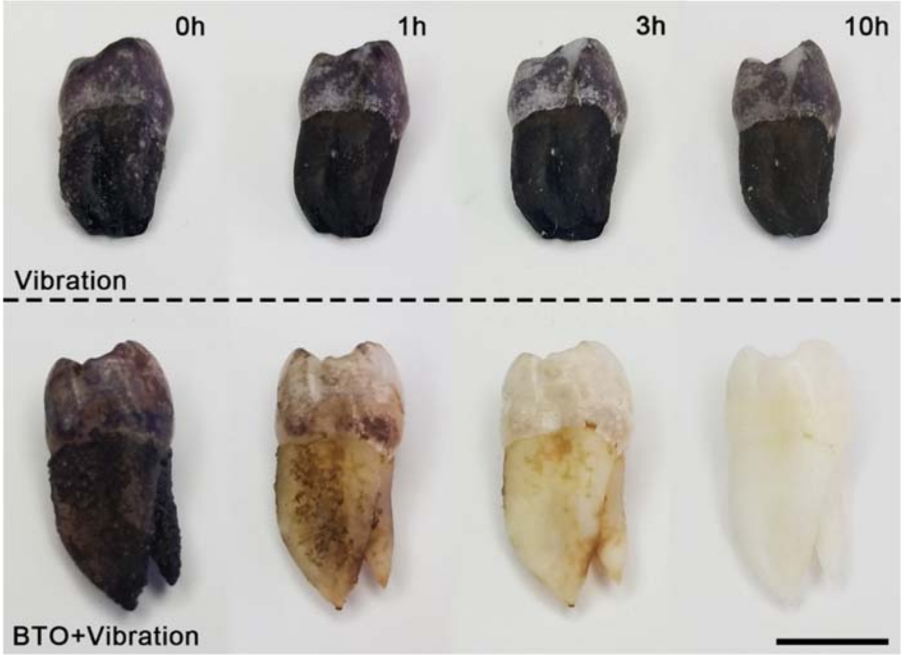

Supplementary Fig. 8 Photographs of teeth stained by red wine under treatment of vibration in (top) pure deionized water and (bottom) turbid liquid of BTO nanoparticles for 0, 1, 3 and 10 hours, respectively. These photographs are successive images of the same tooth. Scale bar is 1 cm.

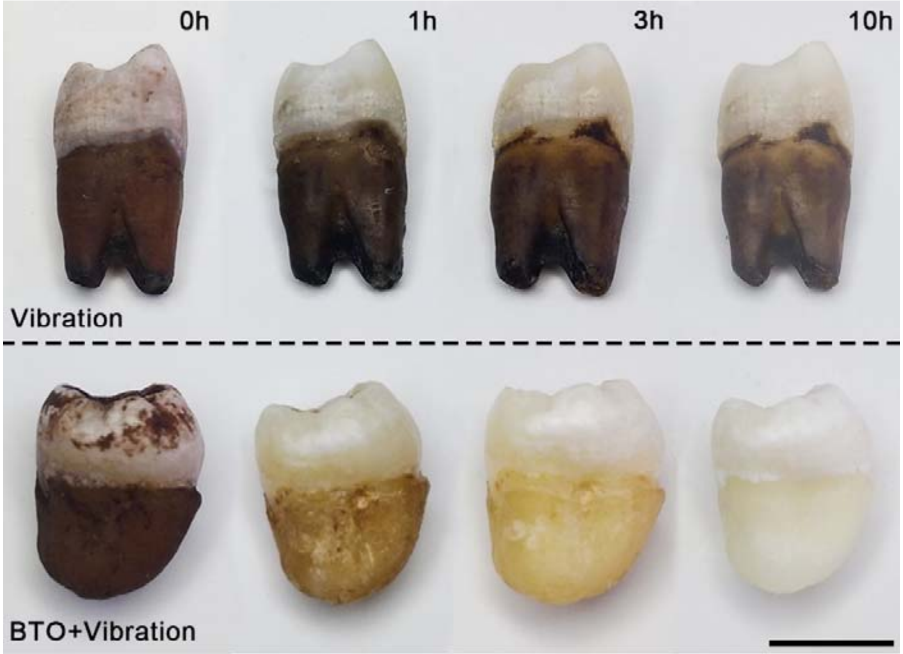

Supplementary Fig. 9 Photographs of teeth stained by blueberry juice under treatment of vibration in (top) pure deionized water and (bottom) turbid liquid of BTO nanoparticles for 0, 1, 3 and 10 hours, respectively. These photographs are successive images of the same tooth. Scale bar is 1 cm.

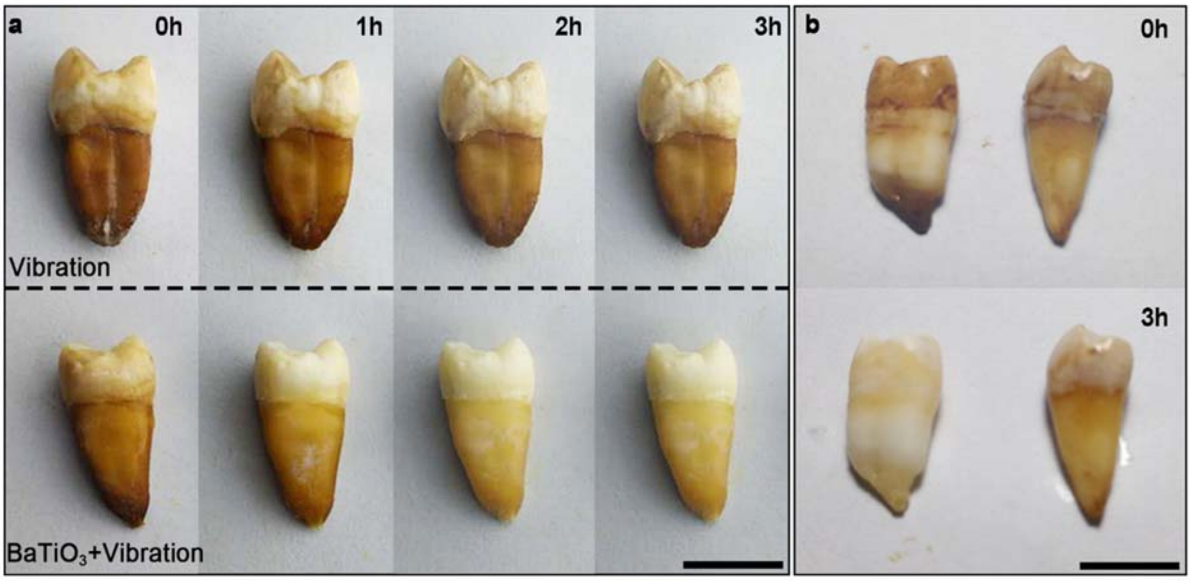

Supplementary Fig. 10 a Photographs of teeth stained by vinegar under treatment of vibration in (top) pure deionized water and (bottom) turbid liquid of BTO nanoparticles for 0, 1, 2 and 3 hours, respectively. These photographs were taken for an identical tooth every one hours. **b** Photographs for the teeth under consecutive vibration for three hours. The left tooth was whitened in turbid liquid of BTO nanoparticles, and the right teeth was in the pure deionized water as the control. Scale bars are 1 cm.

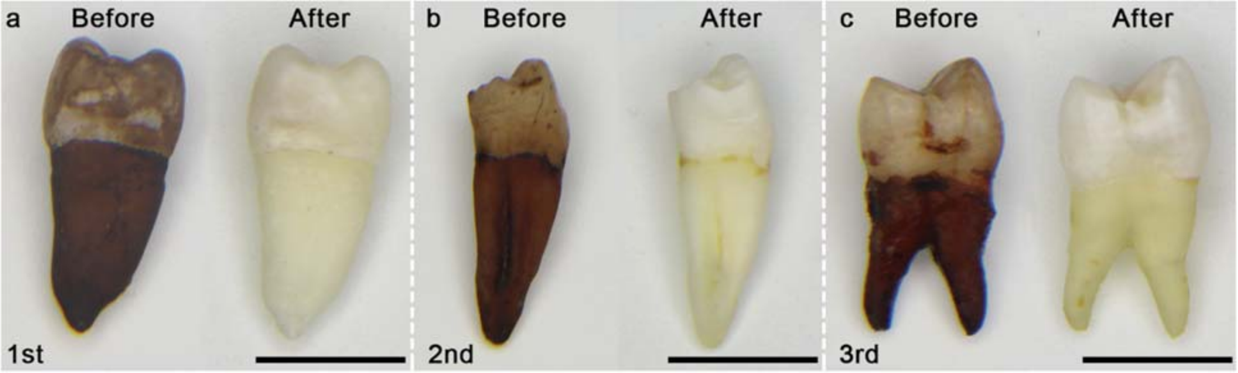

Supplementary Fig. 11 The stability of poled BTO based piezo-catalysis system for tooth whitening. The tooth was vibrated for 10 h in BTO suspension. Scale bars are 1cm.

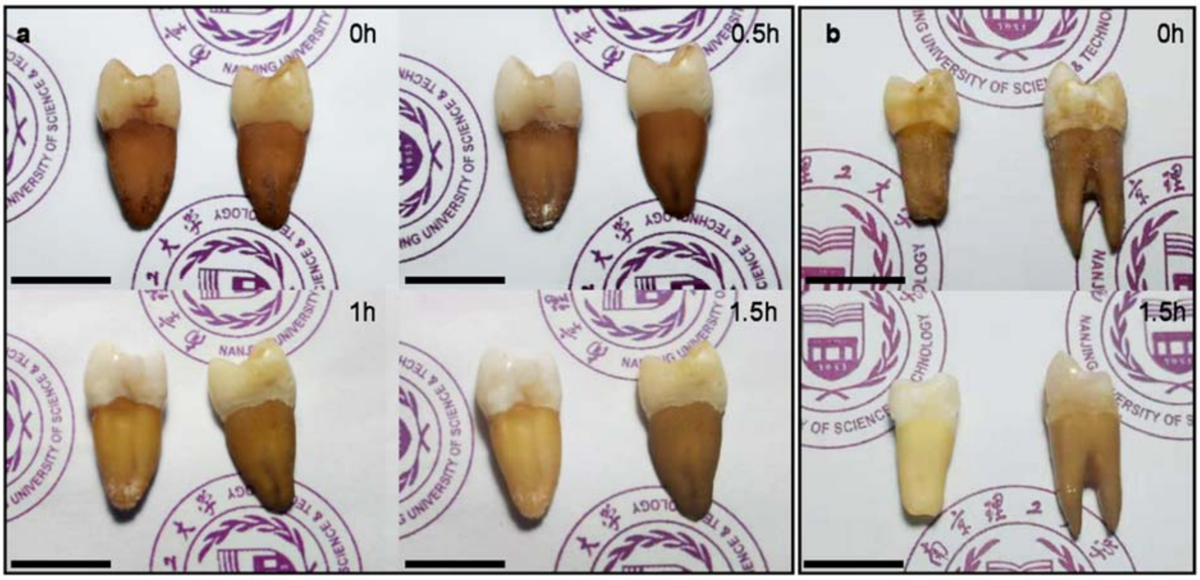

Supplementary Fig. 12 a Photographs for the teeth stained by vinegar under vibration in deionized water (right) and turbid liquid of poled PMN-PT single crystal stones (left) for 0, 0.5, 1 and 1.5 hours, respectively. These photographs were taken with an identical tooth every 0.5 hour for each condition. **b** Photographs for the teeth under consecutive vibration for 1.5 hours. The left tooth was whitened in turbid liquid of poled PMN-PT single crystal stones, and the right teeth was in the pure deionized water as the control. Scale bars are 1 cm.

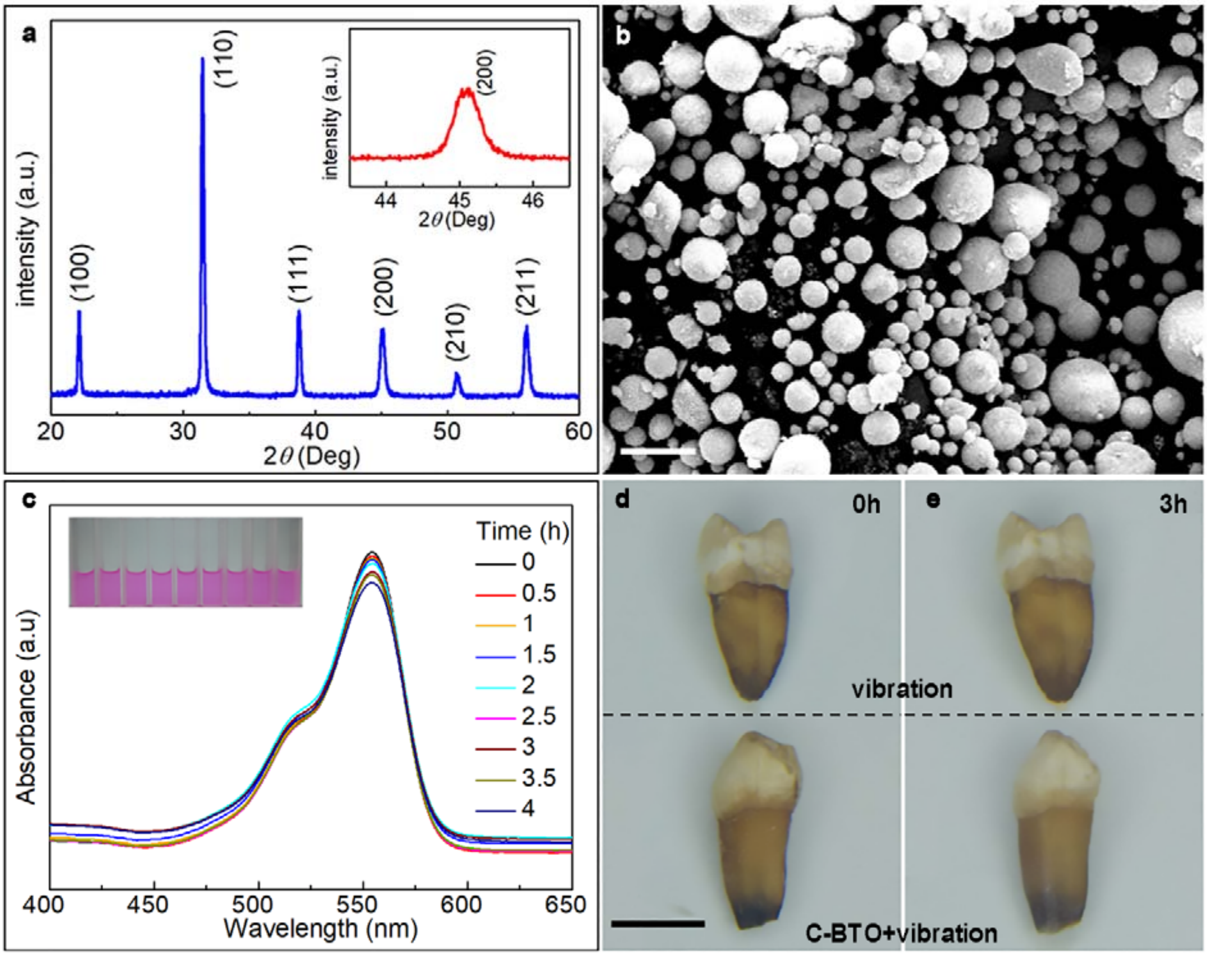

Supplementary Fig. 13 a The XRD data shows no splitting along $(002)_C$ diffraction peak, indicating that the BTO particle is cubic structure without ferroelectricity, **b** the SEM image shows the size of the nanoparticles. **c** There is no change when the cubic BTO was used as catalyst to degrade the RhB solution. For the tooth whitening test, the tooth **d** before vibration and **e** after vibration for 3 hours in turbid liquid of C-BTO (bottom), shows no differences with vibration in deionized water (top) under an identical condition. Scale bar: b is 100 nm, d is 1 cm.

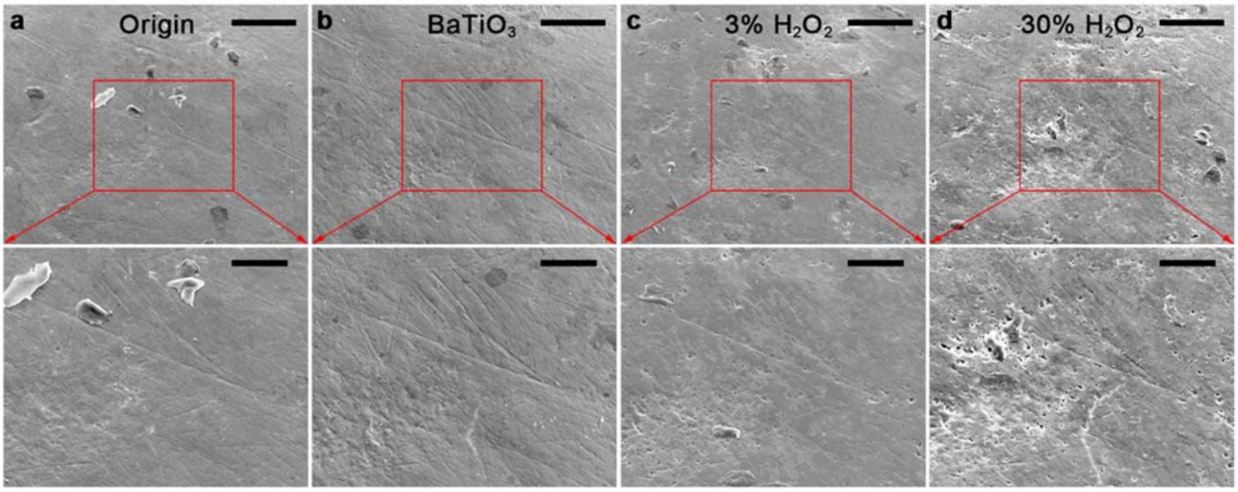

Supplementary Fig. 14 Nondestructive characterization. The scanning electron microscope images of an identical tooth **a** before whitening treatment, **b** after piezo-catalysis whitening in BTO turbid liquid for 10 hours, after further whitening by **c** 3% H_2O_2 for 10 hours and **d** 30% H_2O_2 for 2 hours. The images at the bottom are the enlarged view of the marked identical zone. Scale bars are 100 µm (top) and 50 µm (bottom).

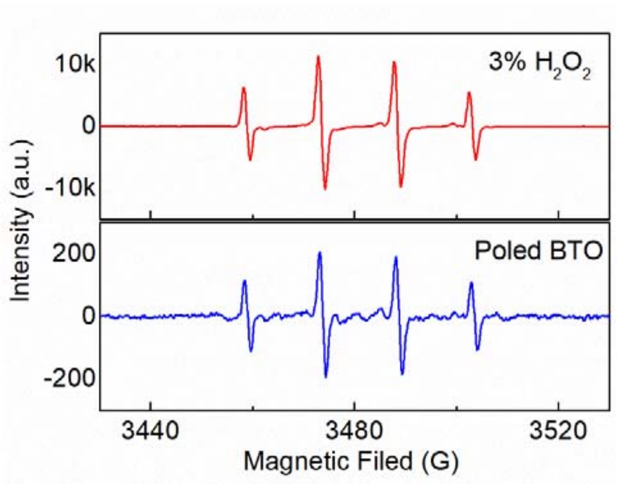

Supplementary Fig. 15 The EPR spectrum of DMPO- •OH created by Fenton reaction in 3% H_2O_2 (top) and poled BTO after vibrated for 5 min (bottom).

It is well known that 3% H_2O_2 is widely used medically to clean mouth, here the concentration of reactive species in 3% H_2O_2 was used as a benchmark of safety. In order to identify the safety of BTO for tooth whitening, we compared the concentration of •OH created by BTO after vibrated for 5 min and that created by Fenton reaction in 3% H_2O_2. The concentration of •OH in 3% H_2O_2 is about 50 times than that created by BTO after vibrated for 5 min. This is further evidence the BTO nanoparticles used for tooth whitening have no risk of excessive free radicals.

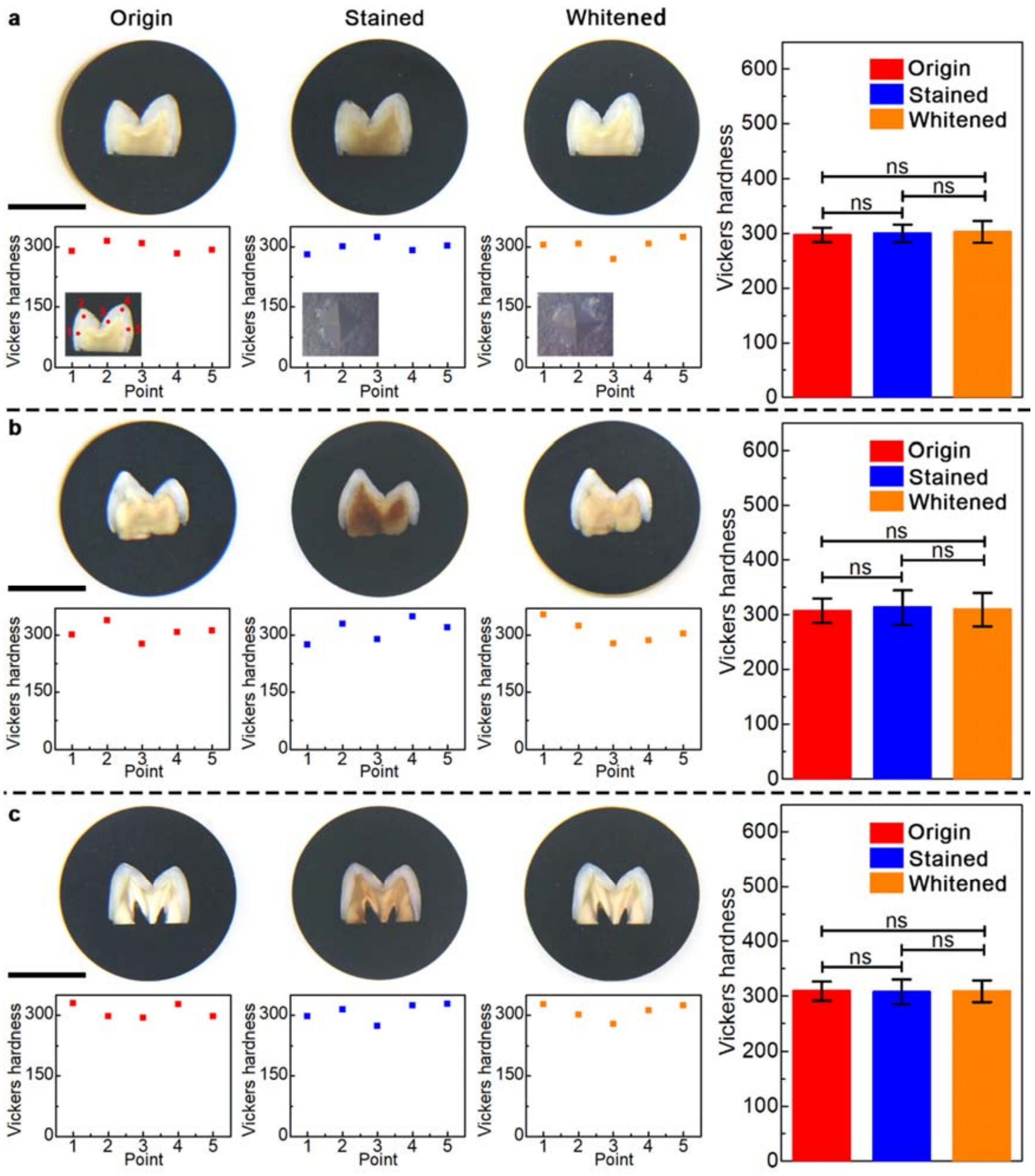

Supplementary Fig. 16 Vickers microhardness test using three different samples, **a** sample 1, **b** sample 2, **c** sample 3. The inset shows the position we test the hardness and the typical Vickers indentation we got. The Vickers hardness of enamel shows no change in all samples. (NS = $p > 0.05$; ANOVA). Scale bars are 1 cm. Error bars = standard deviation (n=5).

Three teeth were randomly selected. Each tooth was cut into two pieces along the buccolingual division line using a line cutting machine. The teeth were then mounted in self-setting polymeric resin at

room temperature. The samples were ground into a flat enamel surface successively using 600, 1000, 1200 grit silicon carbide paper, then polished with 0.2 and 0.05 microns alumina slurry. In order to avoid local overheating on enamel surface, both grinding and polishing procedures were performed in water. Hardness was measured prior to staining, after staining and after whitening using a Vickers microhardness tester (Shanghai Huiju, HVS-1000Z) under a load of 200 g with 10 s dwell time. As shown in Supplementary Fig. 12, we choose five points on the enamel at different position, the hardness of tooth is about 300HV, and the value at different position is similar, which means the tooth is complete and healthy. Most importantly, the hardness shows no change at each step during the whole test, this proved no change in microstructure of the tooth.

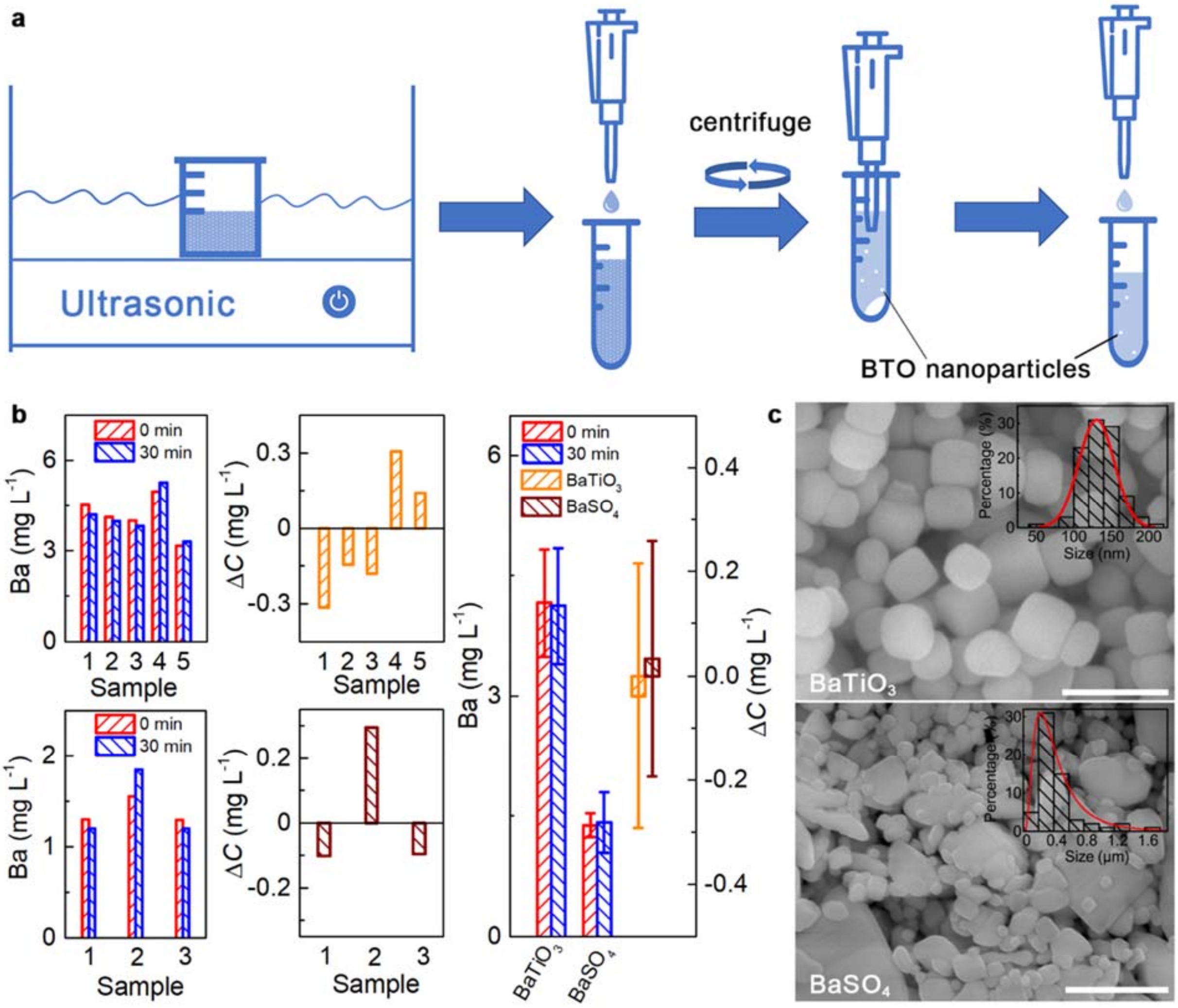

Supplementary Fig. 17 a Schematic diagram of experimental process, **b** (left column) the concentration of total Ba element (including Ba^{2+} and compound) in the supernatant obtained from BTO (top) and $BaSO_4$ (bottom) suspensions before (red) and after (blue) vibration for 30 min; (middle column) the Ba element concentration difference of before-vibration and after-vibration for 30 min; (right column) concentration of total Ba element and the difference with experimental errors. **c** the SEM image shows particles with different size of BTO (top) and $BaSO_4$ (bottom). Scale bars in c are 200 nm (top) and 500 nm (bottom). Error bars = standard deviation (n=5 for $BaTiO_3$, n=3 for $BaSO_4$).

In order to check the possible leakage of Ba ions during vibration, 50 mg BTO nanoparticles were dispersed in 50 ml deionized water, and the obtained solutions were stirred for 30 min to make sure that the BTO nanoparticles were evenly dispersed. Then, the suspension was vibrated for 30 min (i.e., daily

toothbrush time is less than 30 min). 10 ml suspension was collected at a certain time and centrifuged in order to remove the nanoparticles. The supernatant was collected and this process repeated for three times in order to avoid experimental error. The concentration of barium in the supernatant was measured by Inductively Coupled Plasma-Optical Emission Spectroscopy (ICP-OMS), which is used to measure the concentration of an element, including the existence of related ions and compounds. The barium meal ($BaSO_4$) is often used as gastrointestinal radiological examinations, indicating that $BaSO_4$ is safe, so the same experiment was taken using $BaSO_4$ as comparison. The left column of Supplementary Fig. 17 c shows that the barium element was detected in the supernatant of both BTO and $BaSO_4$ before and after vibration, while the concentration difference of barium element before-and after-vibration for both BTO and $BaSO_4$ supernatant changed randomly (middle column of Supplementary Fig. 17 c). The obtained concentration of Ba element and the difference with experimental errors of before-vibration and after-vibration was given in the right column of Supplementary Fig. 17 c. The existence of Ba element in the supernatant is possible due to the incomplete removal of nanoparticles by centrifugation. Because the concentration of barium element in supernatant of $BaSO_4$ was a little lower than that of BTO, which can be understood by particle size of $BaSO_4$ and BTO (i.e., ~ 300 nm relative to ~ 150 nm). These result in less $BaSO_4$ nanoparticles remains in the supernatant after centrifugation. The comparation of the concentration difference of barium before- and after- vibration between BTO and $BaSO_4$ shows that the barium concentration difference is near zero and experimental error varies within the same range. In turn, we can conclude that the variation in barium concentration before and after vibration was caused by experimental errors rather than the leakage of Ba^{2+}.

1. Park S, *et al.* A ferroelectric photocatalyst for enhancing hydrogen evolution: polarized particulate suspension. *Phys Chem Chem Phys* **16**, 10408-10413 (2014).
