# Supplementary figures and images for: Piezo-catalysis for nondestructive tooth whitening
Source: Nat Commun. 2020 Mar 12;11:1328. doi: 10.1038/s41467-020-15015-3 (PMC7067860; doi:10.1038/s41467-020-15015-3)

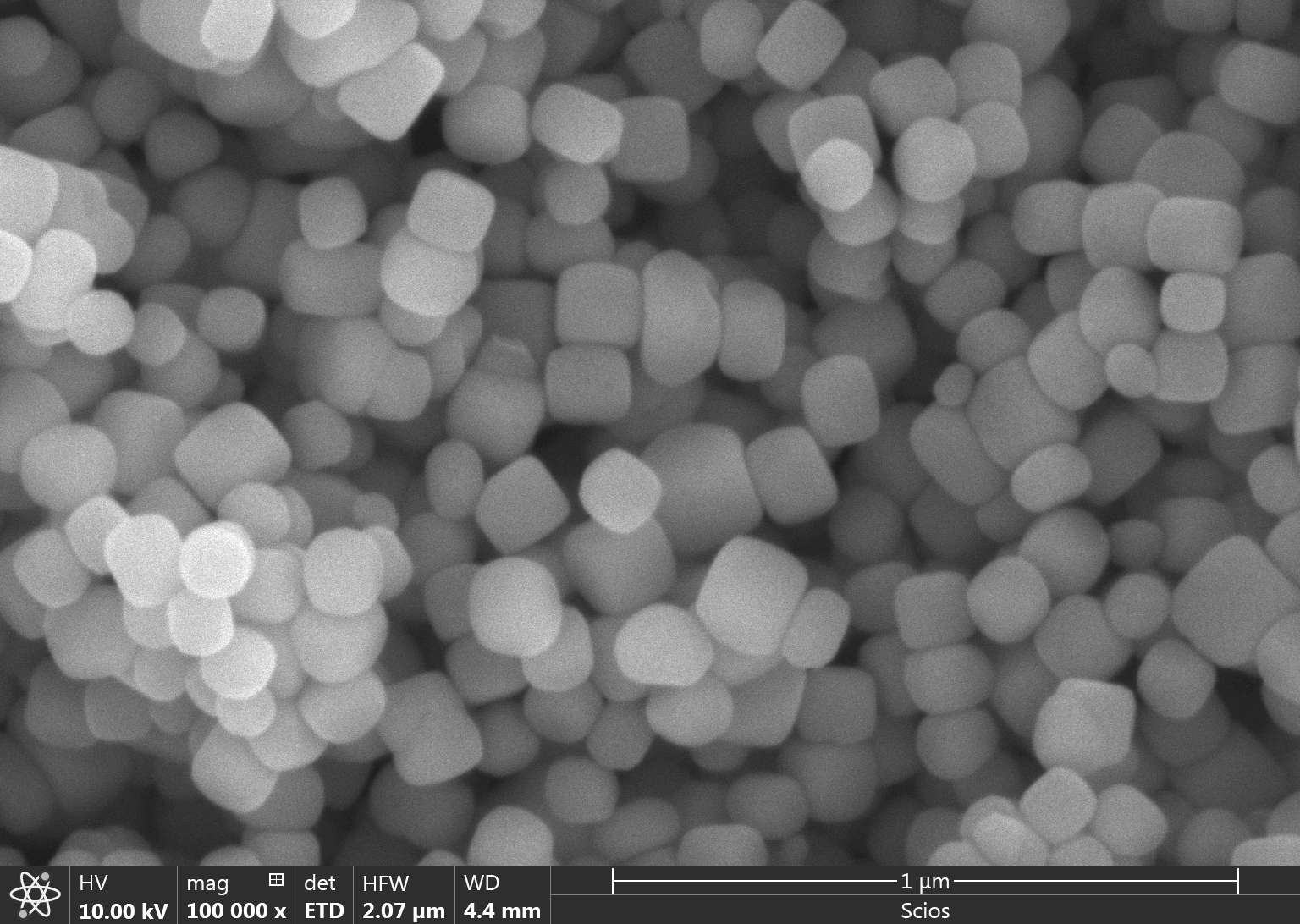

Supplement: Supplementary file 4 — Source Data [file 41467_2020_15015_MOESM4_ESM.zip › Figure 2/Figure 2b.tif]

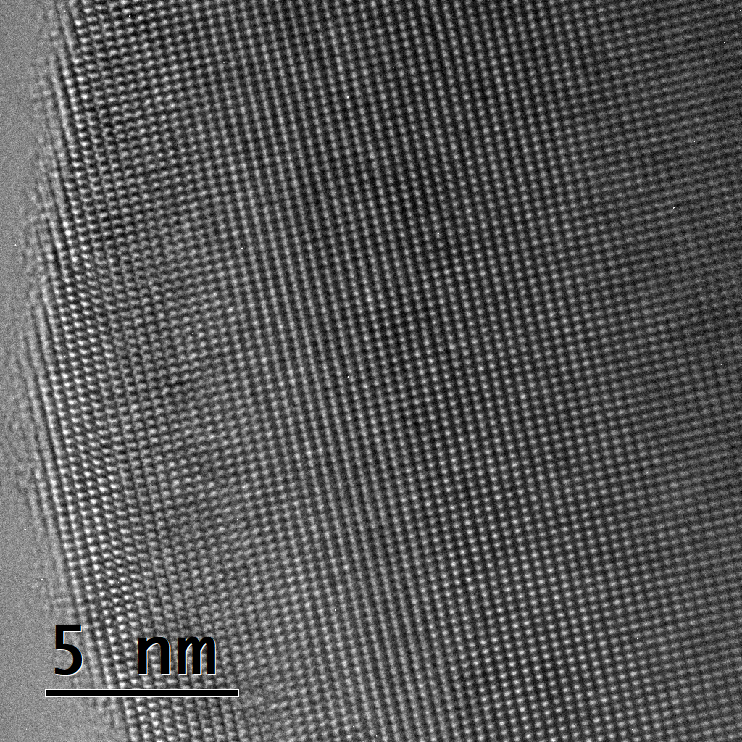

Supplement: Supplementary file 4 — Source Data [file 41467_2020_15015_MOESM4_ESM.zip › Figure 2/Figure 2d.tif]

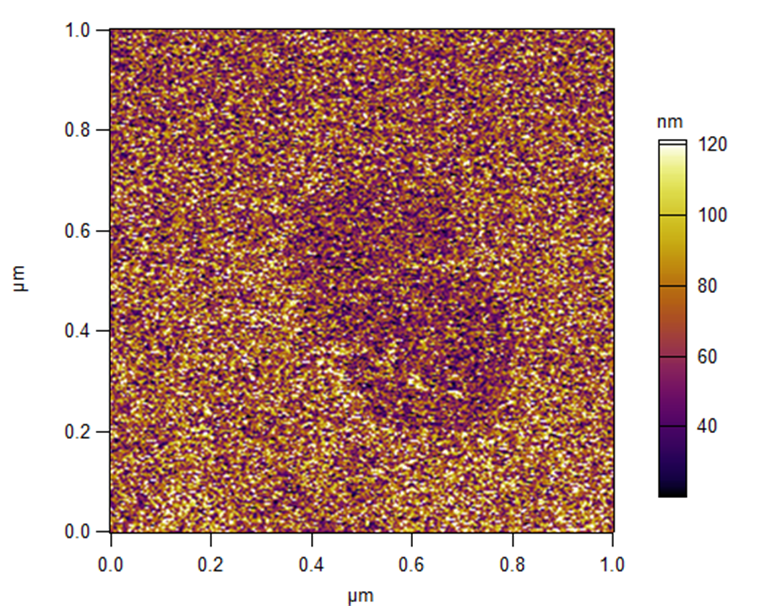

Supplement: Supplementary file 4 — Source Data [file 41467_2020_15015_MOESM4_ESM.zip › Figure 2/Figure 2f.tif]

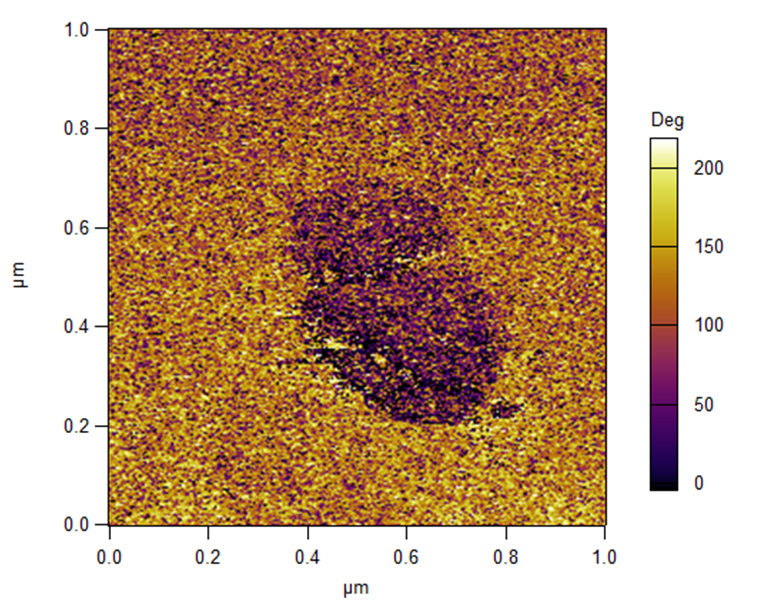

Supplement: Supplementary file 4 — Source Data [file 41467_2020_15015_MOESM4_ESM.zip › Figure 2/Figure 2g.tif]

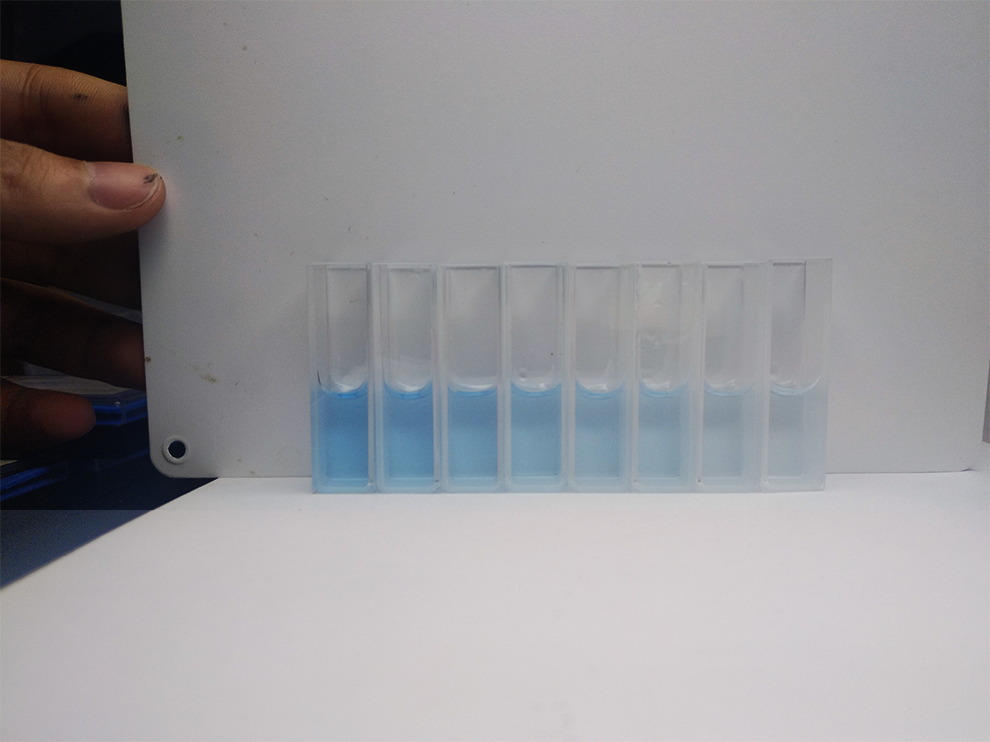

Supplement: Supplementary file 4 — Source Data [file 41467_2020_15015_MOESM4_ESM.zip › Figure 3/Figure 3a inset.tif]

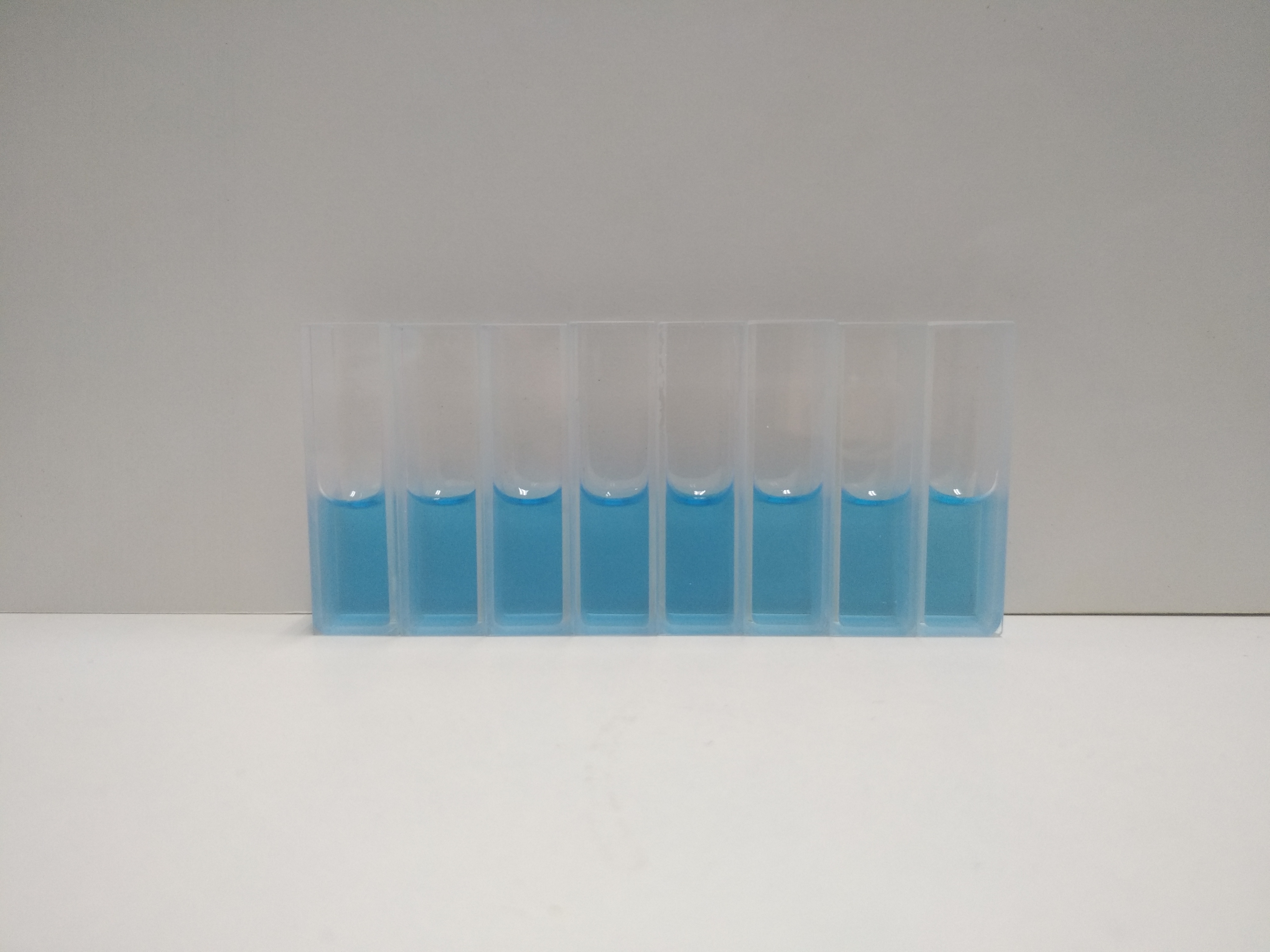

Supplement: Supplementary file 4 — Source Data [file 41467_2020_15015_MOESM4_ESM.zip › Figure 3/Figure 3b inset.jpg]

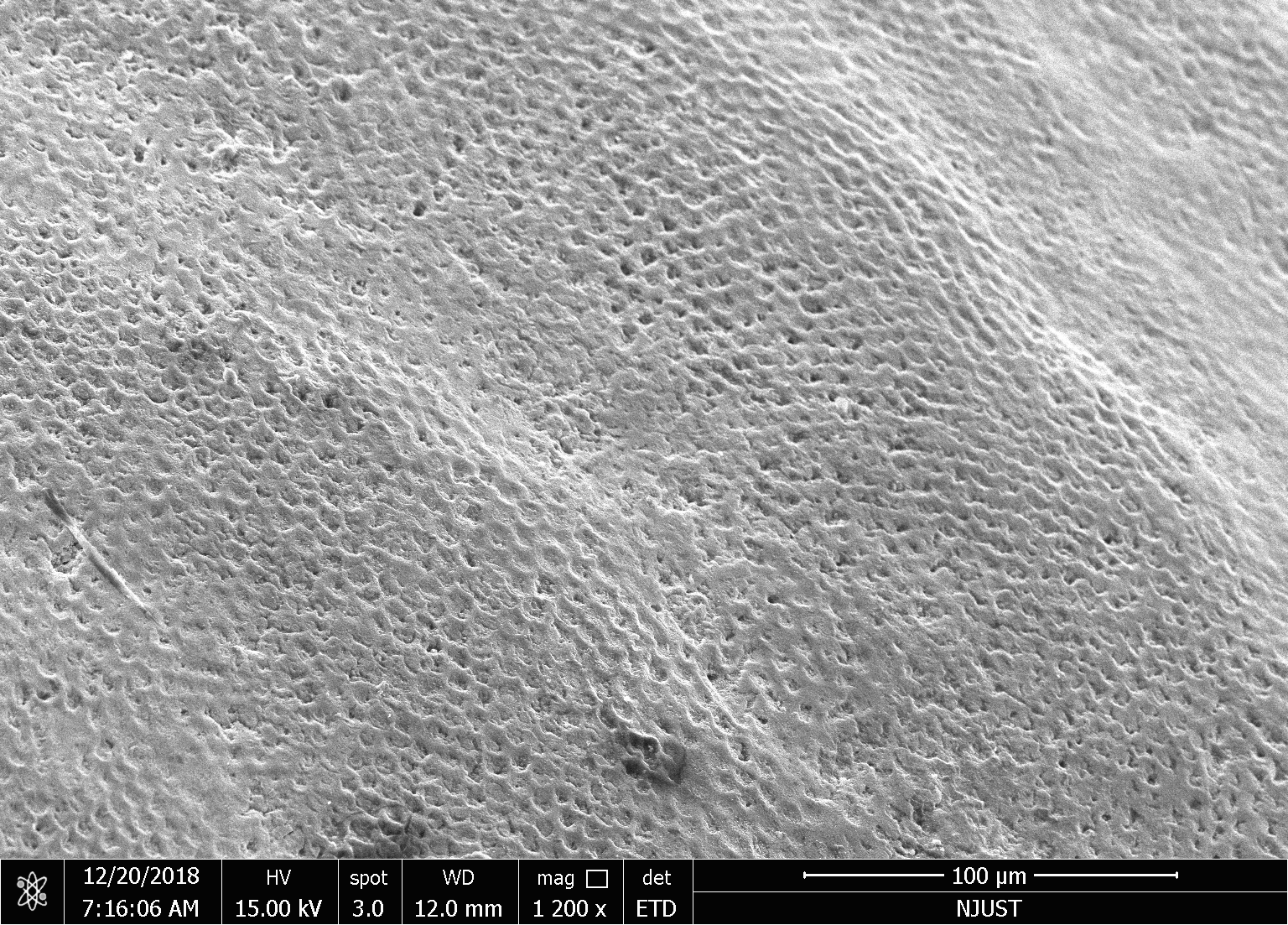

Supplement: Supplementary file 4 — Source Data [file 41467_2020_15015_MOESM4_ESM.zip › Figure 5/Figure 5a-bottom.tif]

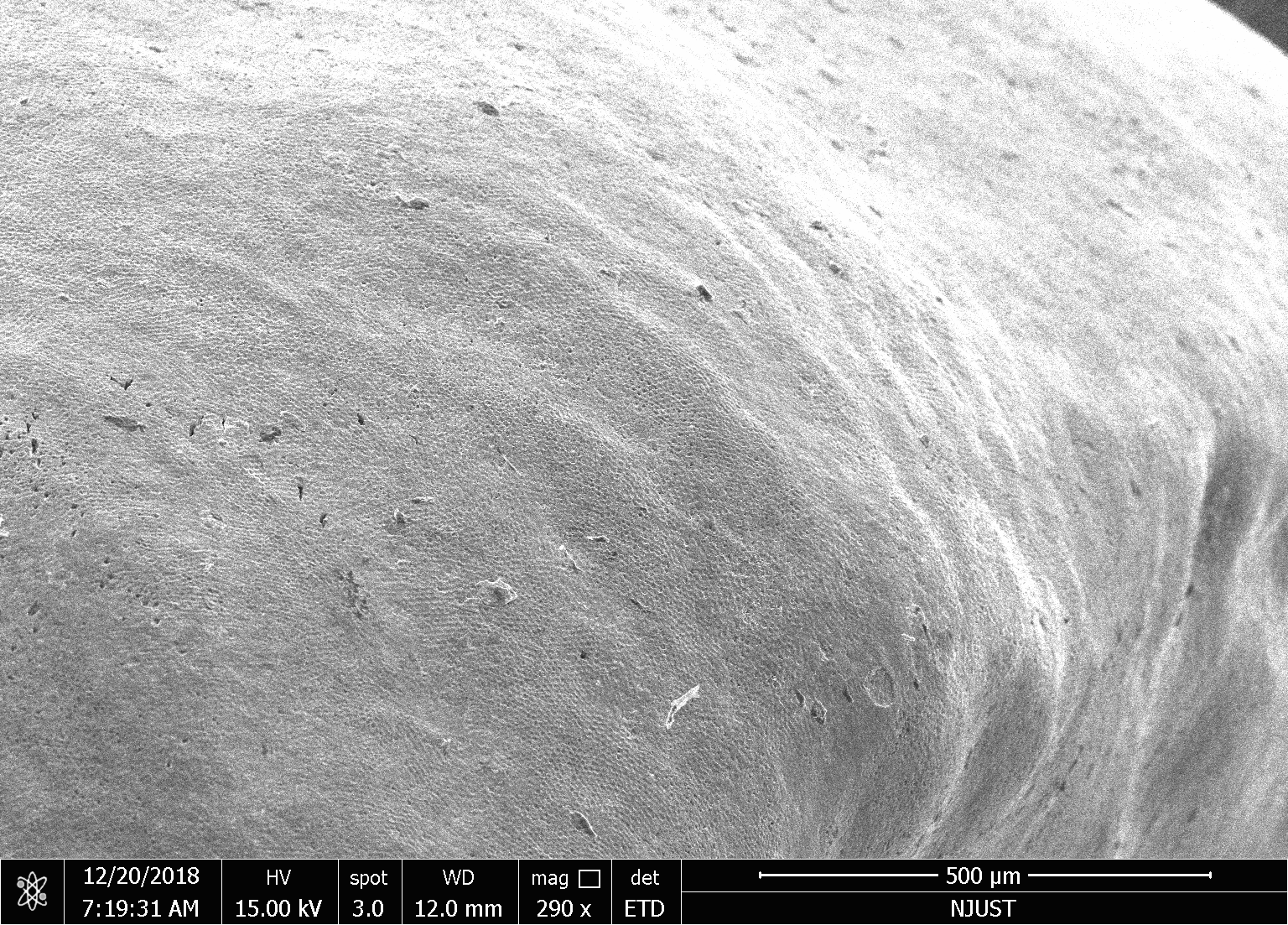

Supplement: Supplementary file 4 — Source Data [file 41467_2020_15015_MOESM4_ESM.zip › Figure 5/Figure 5a.tif]

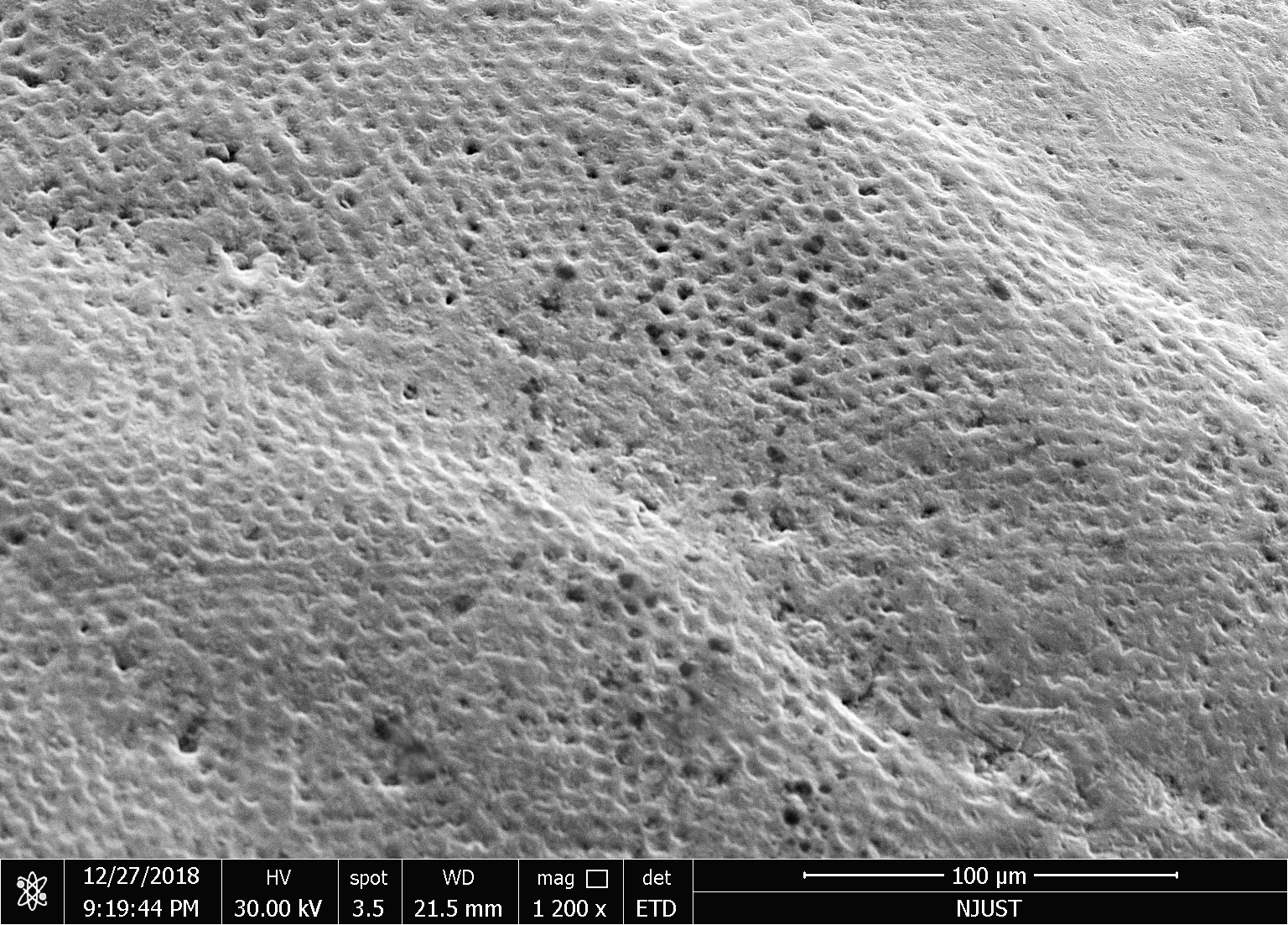

Supplement: Supplementary file 4 — Source Data [file 41467_2020_15015_MOESM4_ESM.zip › Figure 5/Figure 5b-bottom.tif]

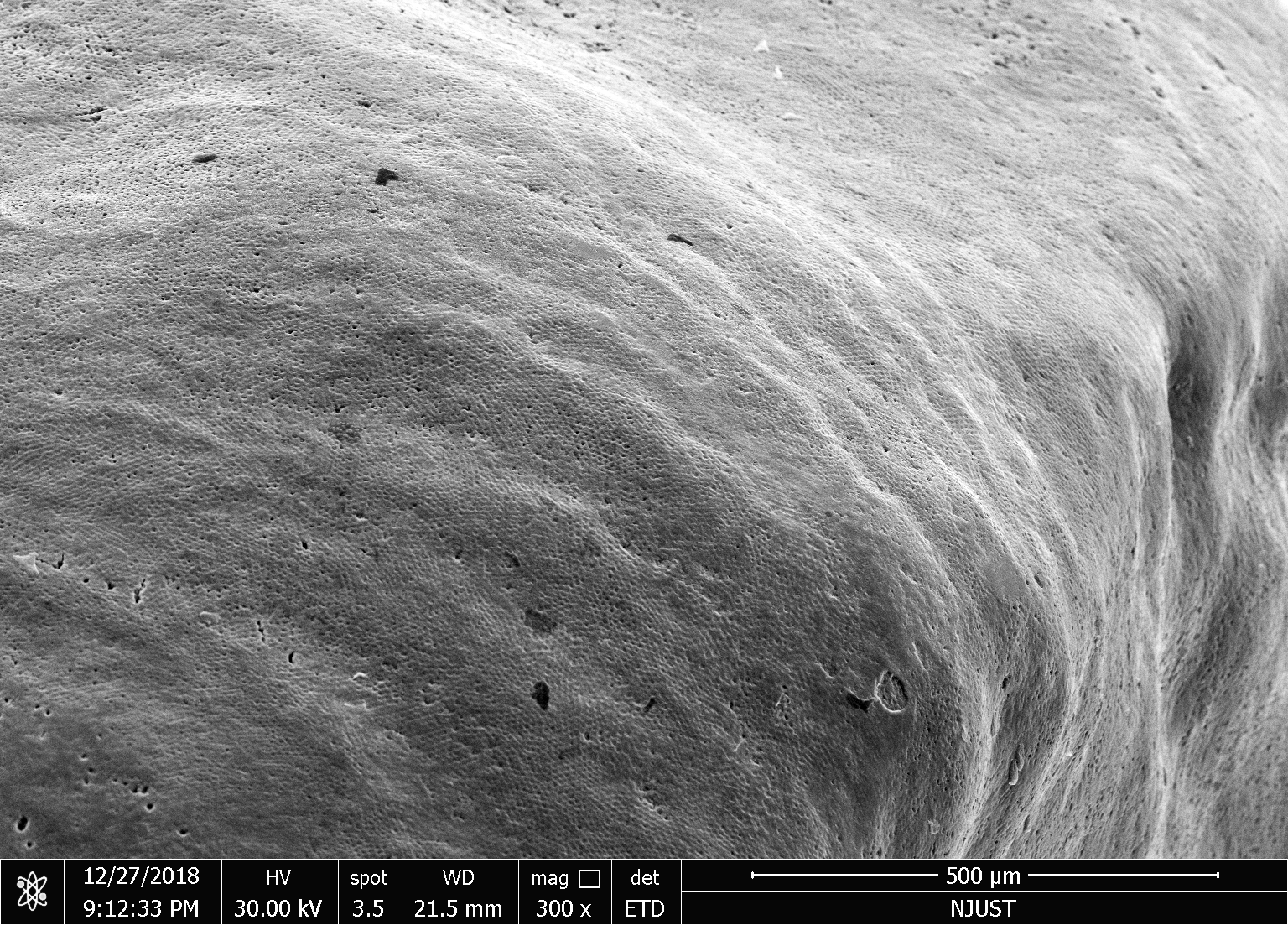

Supplement: Supplementary file 4 — Source Data [file 41467_2020_15015_MOESM4_ESM.zip › Figure 5/Figure 5b.tif]

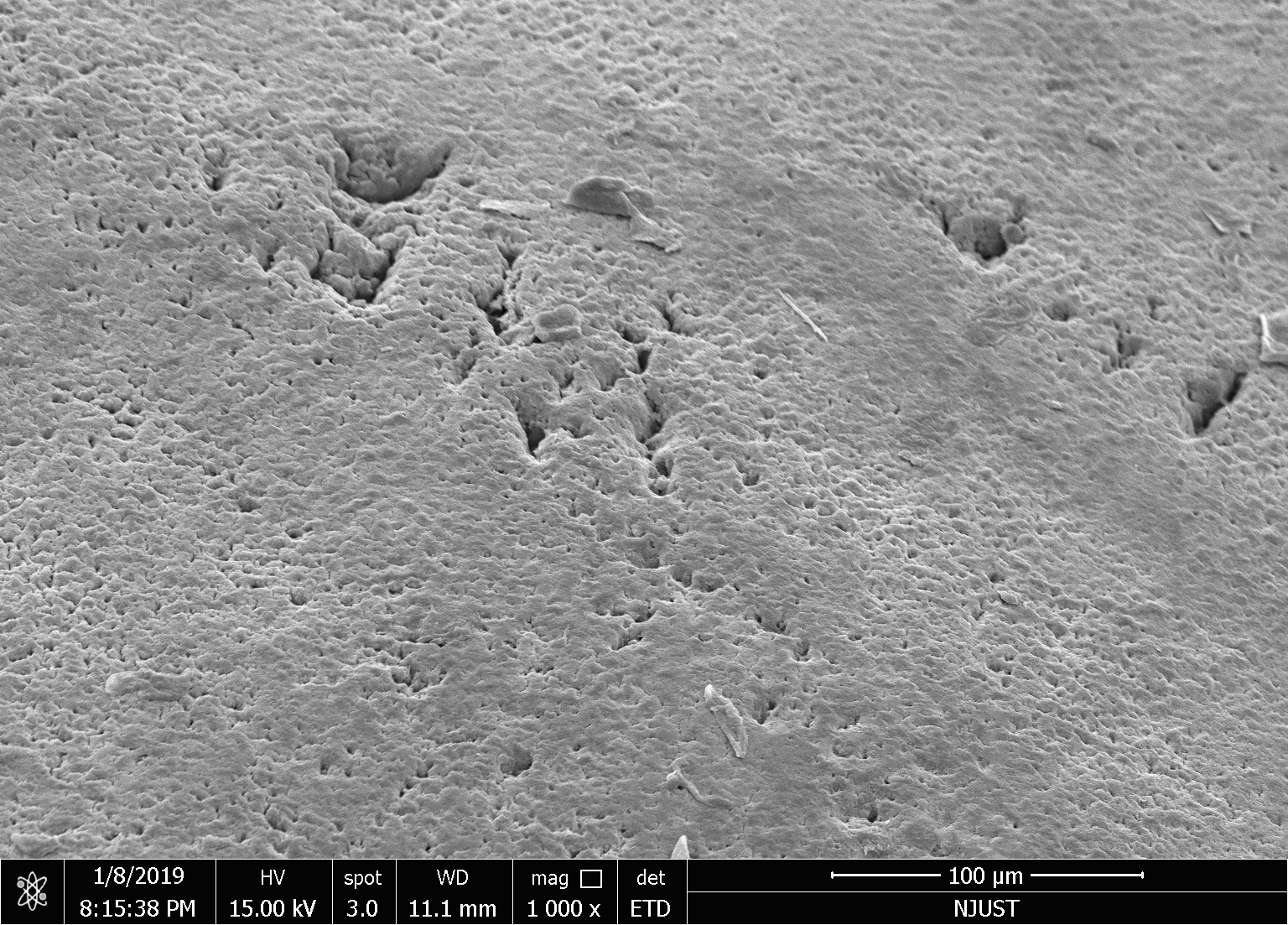

Supplement: Supplementary file 4 — Source Data [file 41467_2020_15015_MOESM4_ESM.zip › Figure 5/Figure 5c-bottom.tif]

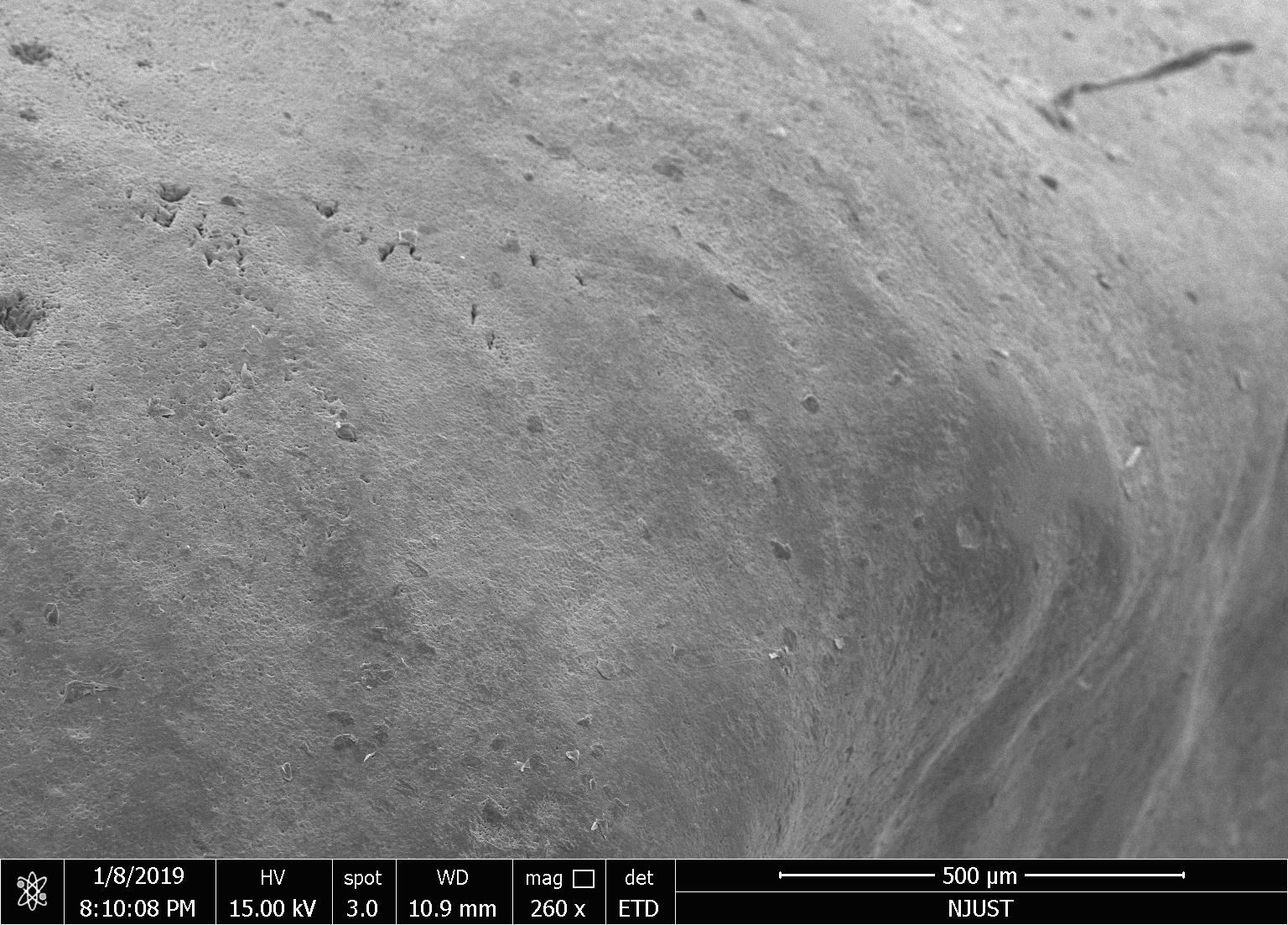

Supplement: Supplementary file 4 — Source Data [file 41467_2020_15015_MOESM4_ESM.zip › Figure 5/Figure 5c.tif]

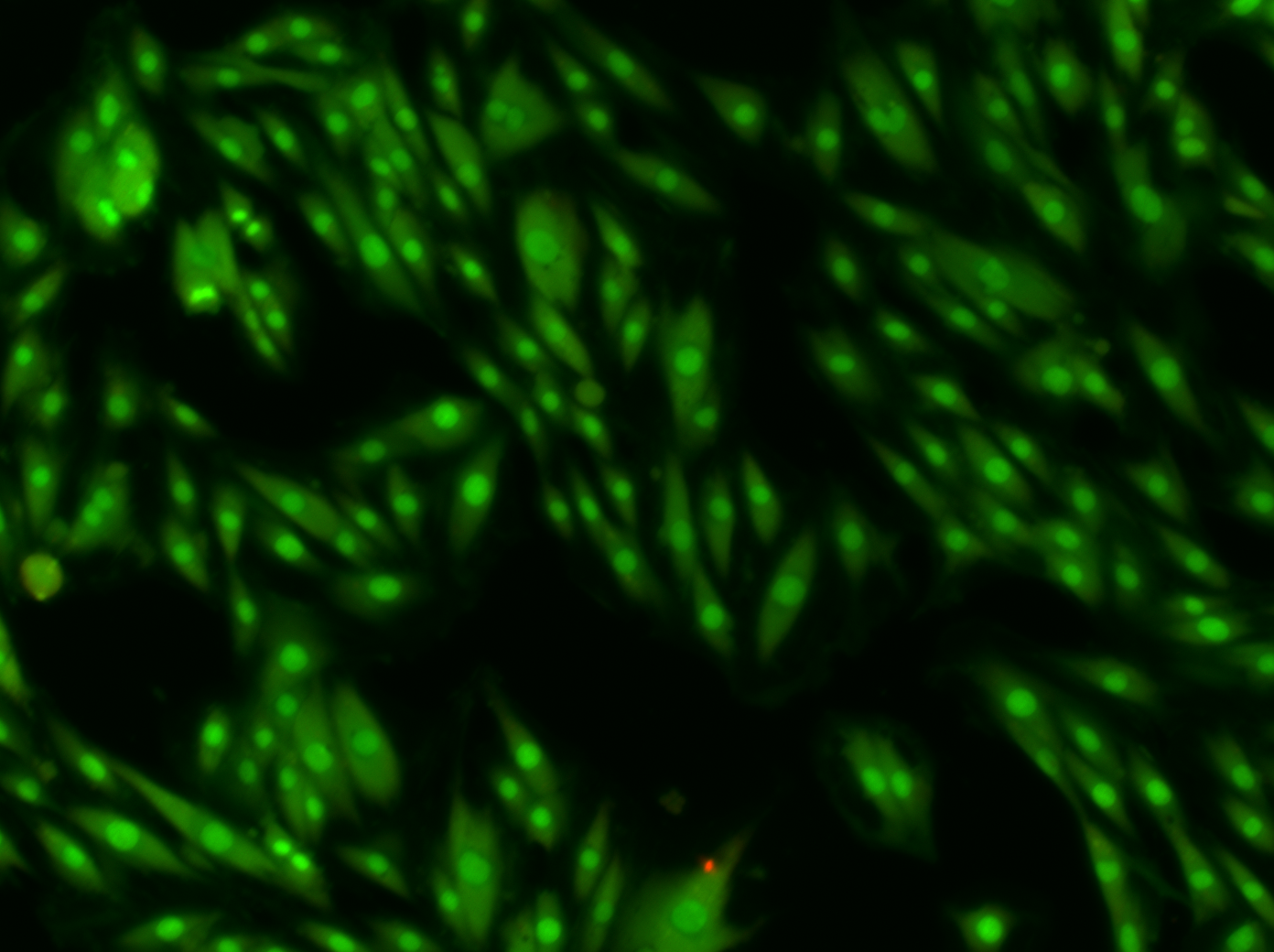

Supplement: Supplementary file 4 — Source Data [file 41467_2020_15015_MOESM4_ESM.zip › Figure 6/Figure 6a-day1.tif]

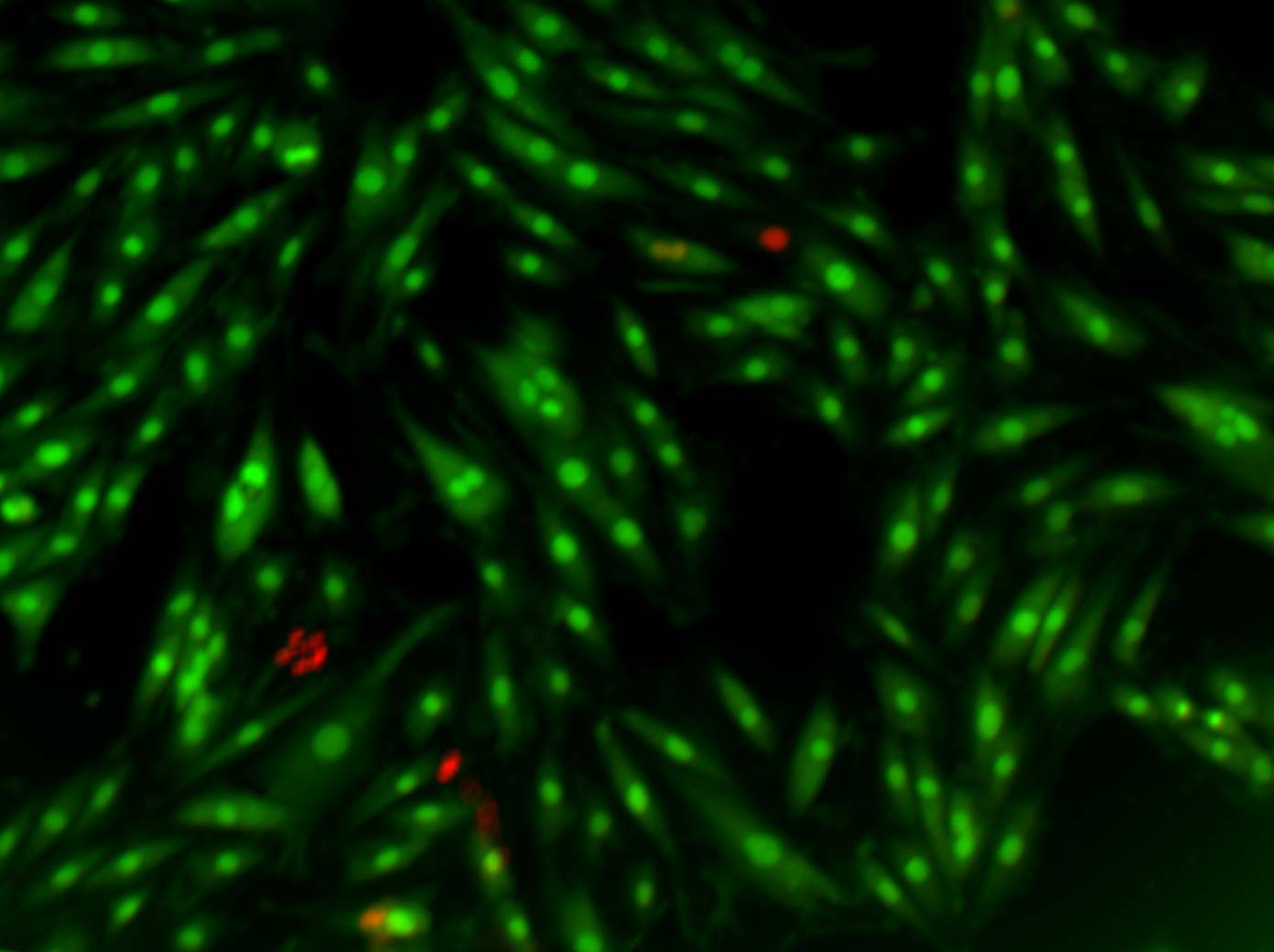

Supplement: Supplementary file 4 — Source Data [file 41467_2020_15015_MOESM4_ESM.zip › Figure 6/Figure 6a-day2.tif]

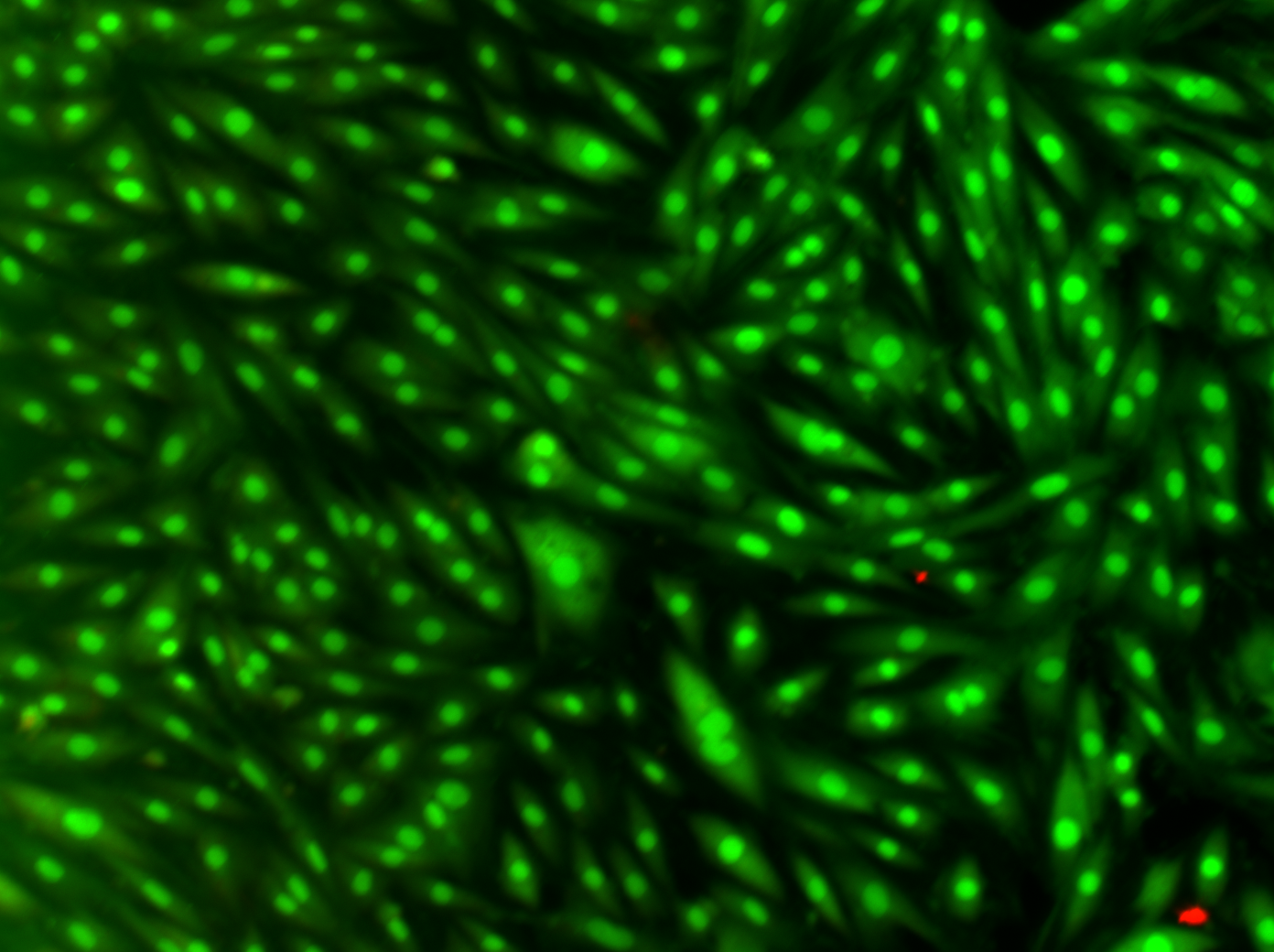

Supplement: Supplementary file 4 — Source Data [file 41467_2020_15015_MOESM4_ESM.zip › Figure 6/Figure 6a-day3.tif]

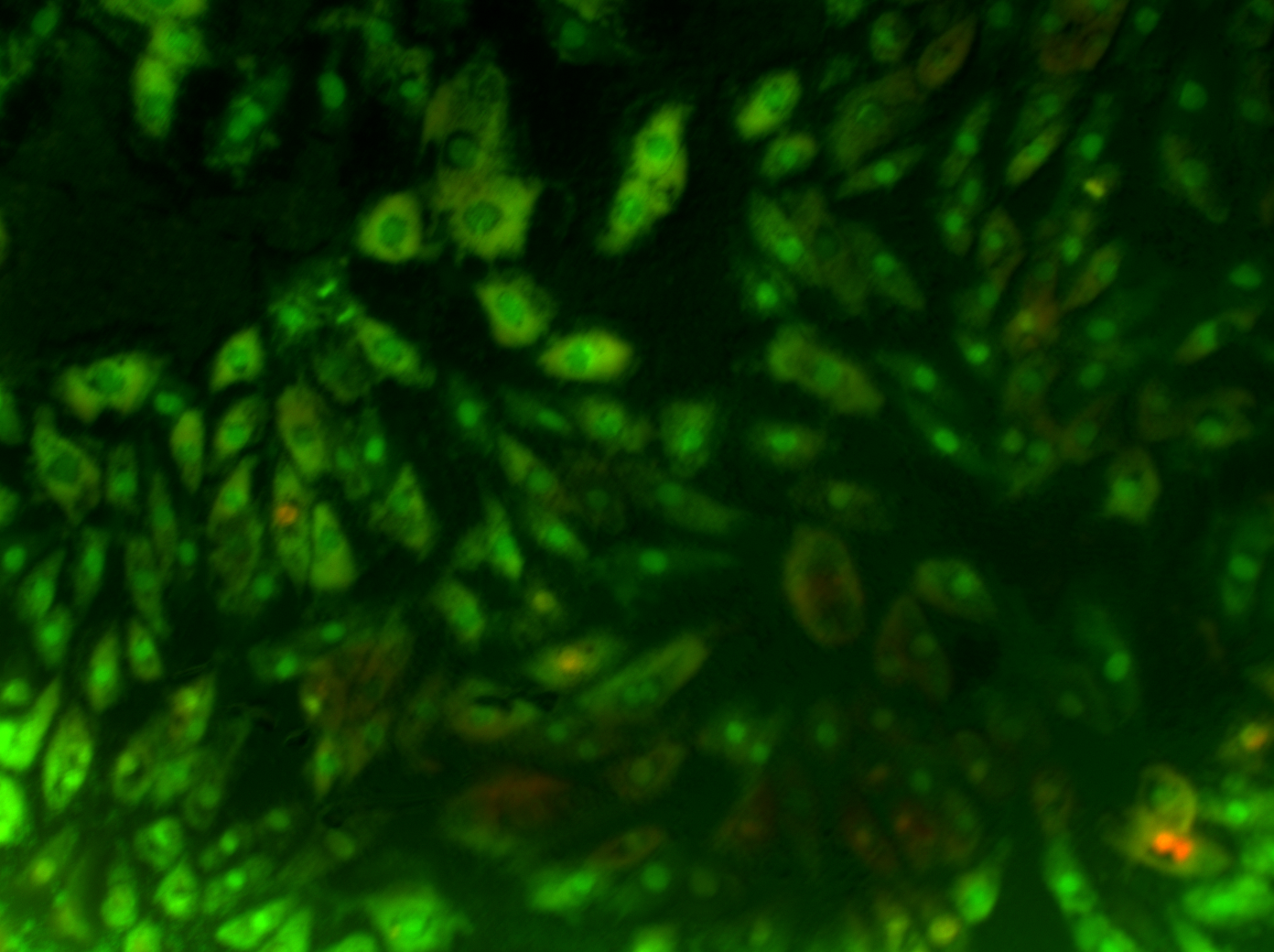

Supplement: Supplementary file 4 — Source Data [file 41467_2020_15015_MOESM4_ESM.zip › Figure 6/Figure 6b-day1.tif]

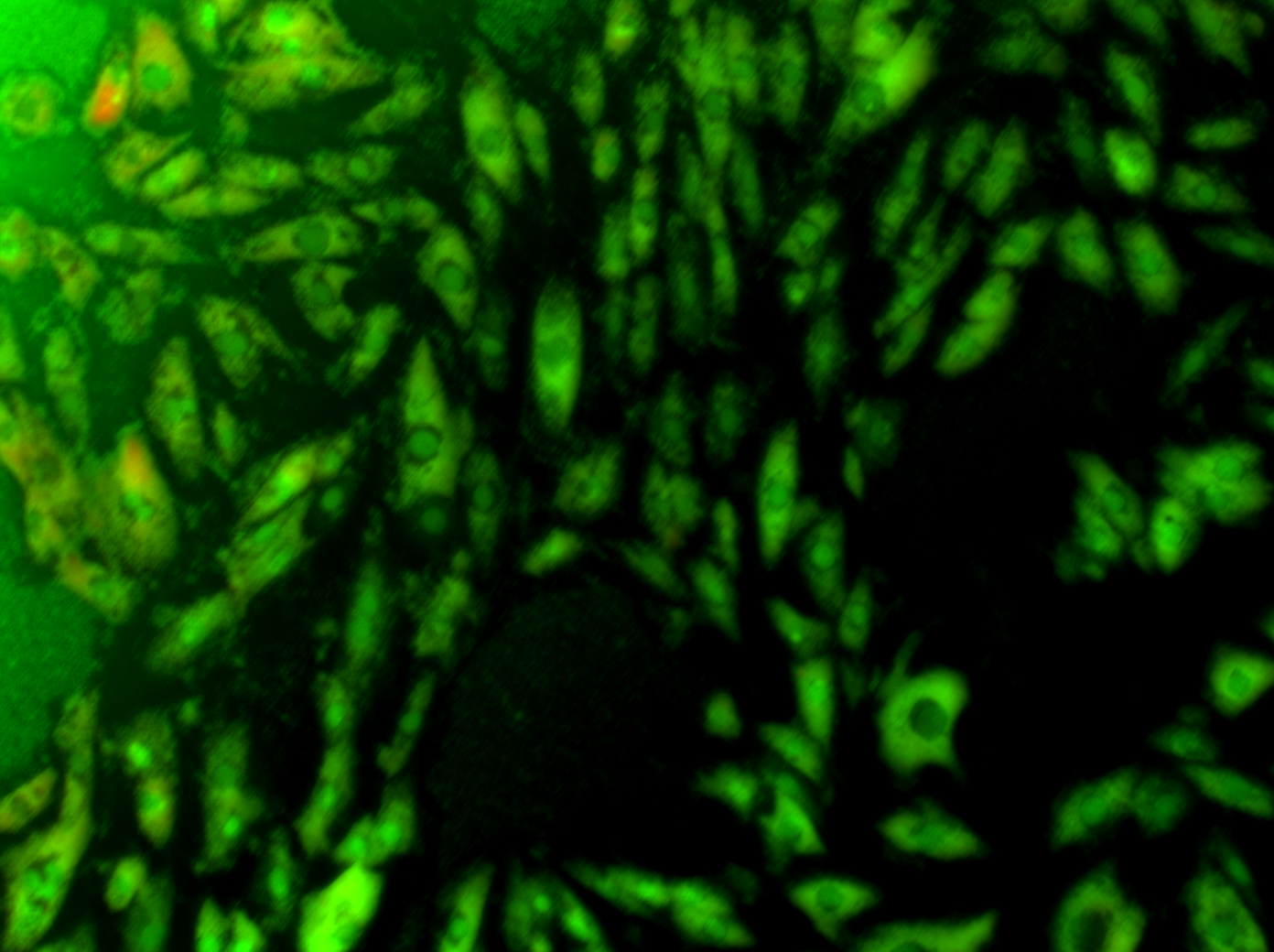

Supplement: Supplementary file 4 — Source Data [file 41467_2020_15015_MOESM4_ESM.zip › Figure 6/Figure 6b-day2.tif]

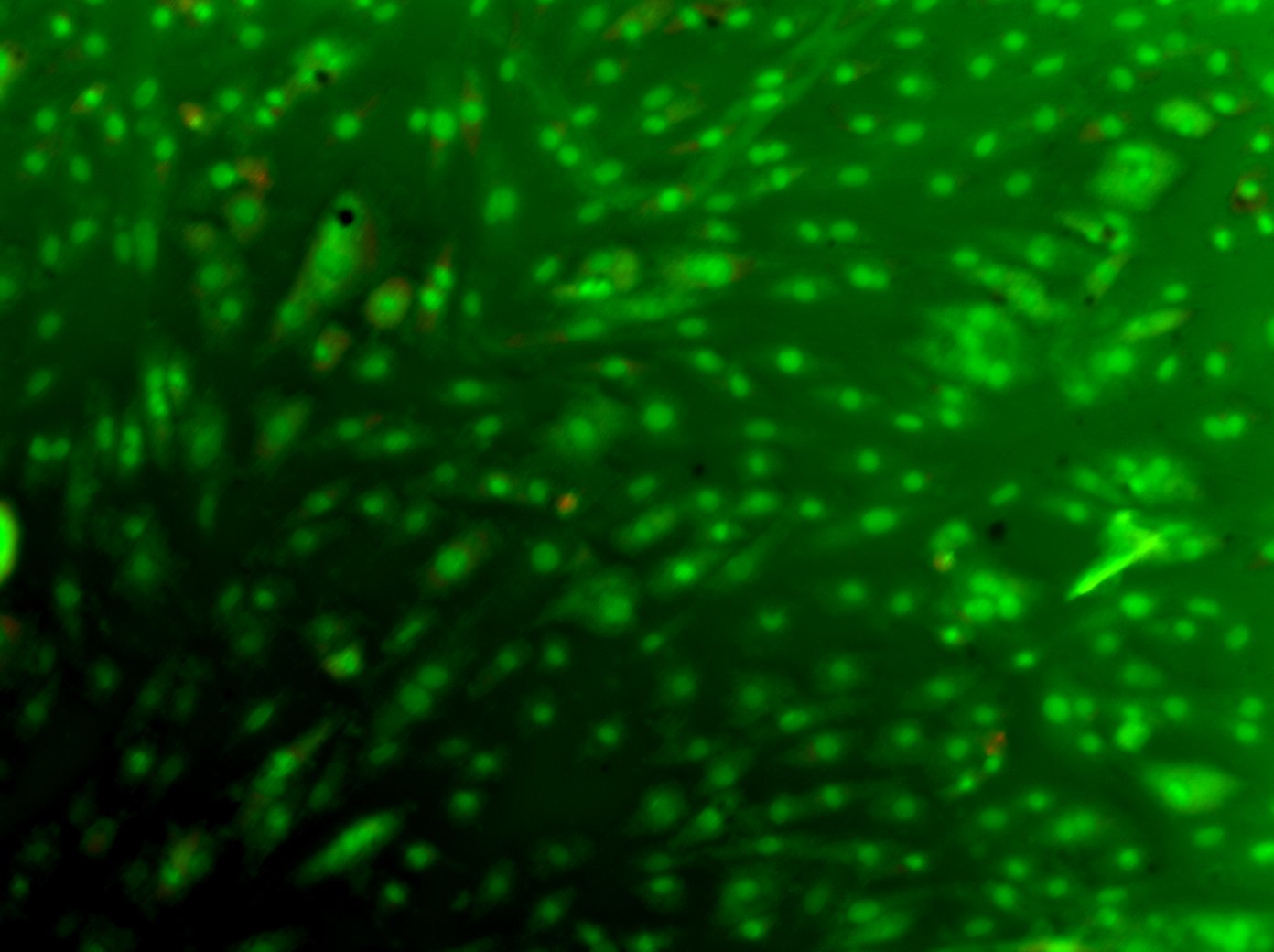

Supplement: Supplementary file 4 — Source Data [file 41467_2020_15015_MOESM4_ESM.zip › Figure 6/Figure 6b-day3.tif]

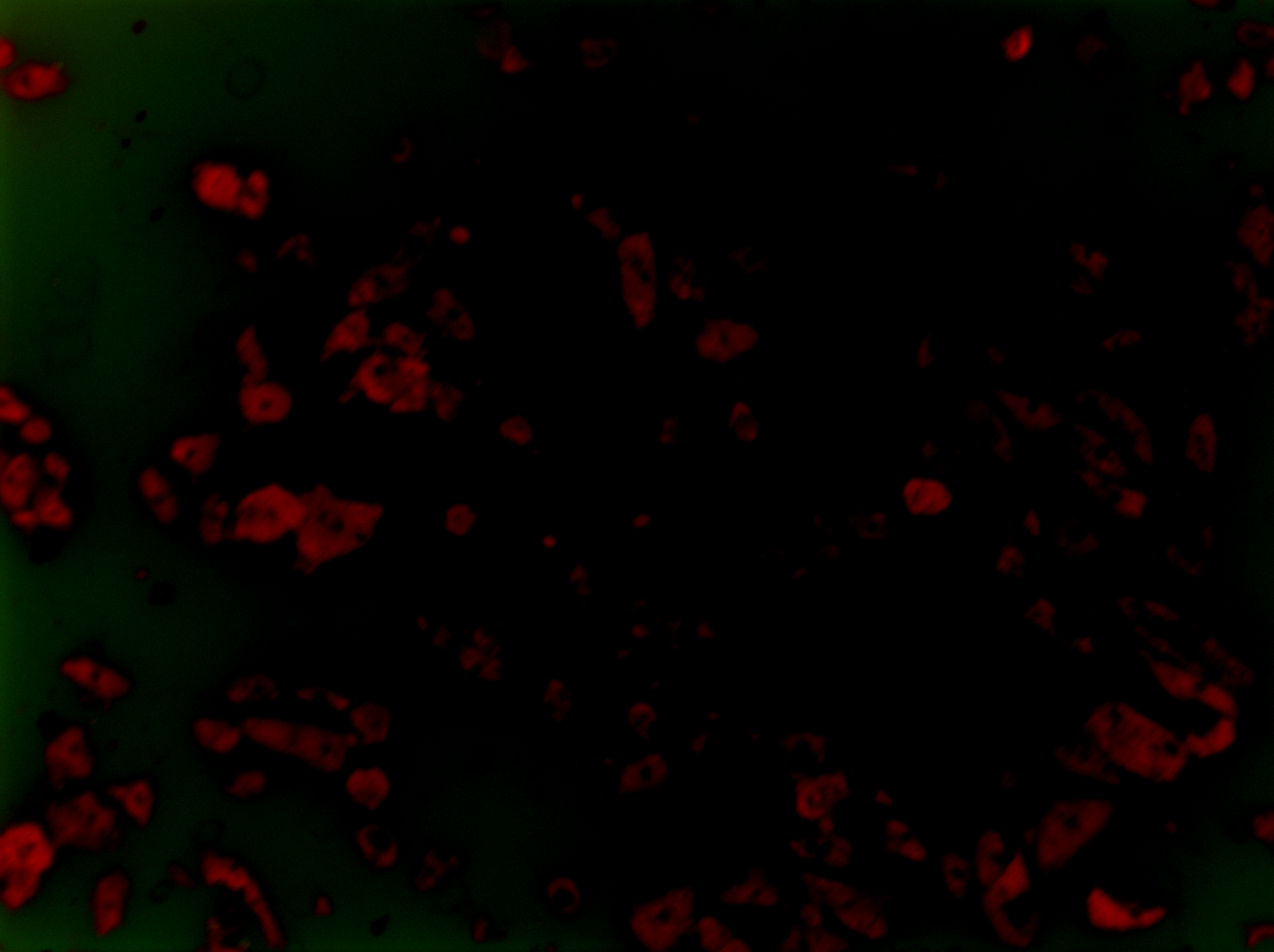

Supplement: Supplementary file 4 — Source Data [file 41467_2020_15015_MOESM4_ESM.zip › Figure 6/Figure 6c-day1.tif]

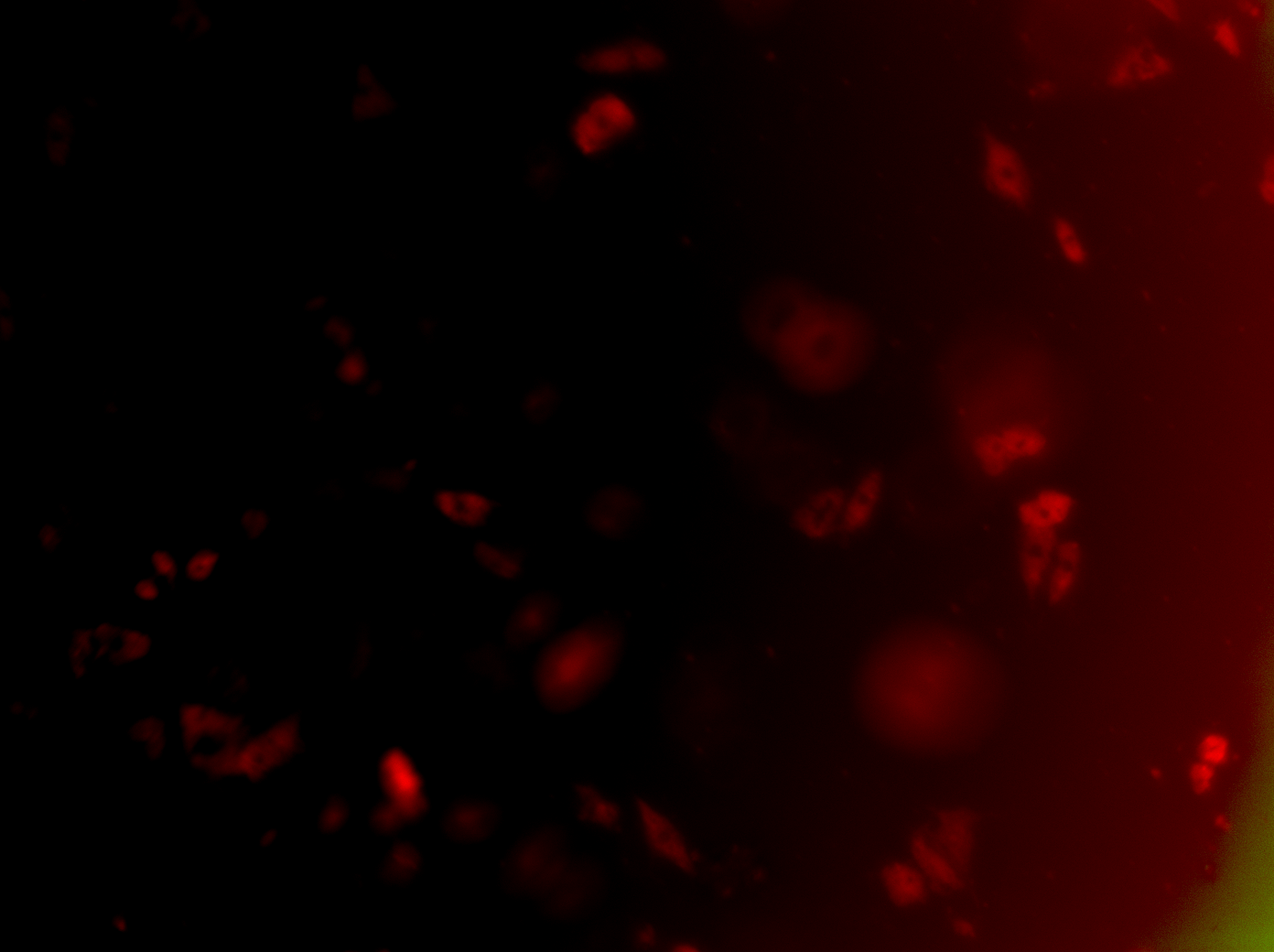

Supplement: Supplementary file 4 — Source Data [file 41467_2020_15015_MOESM4_ESM.zip › Figure 6/Figure 6c-day2.tif]

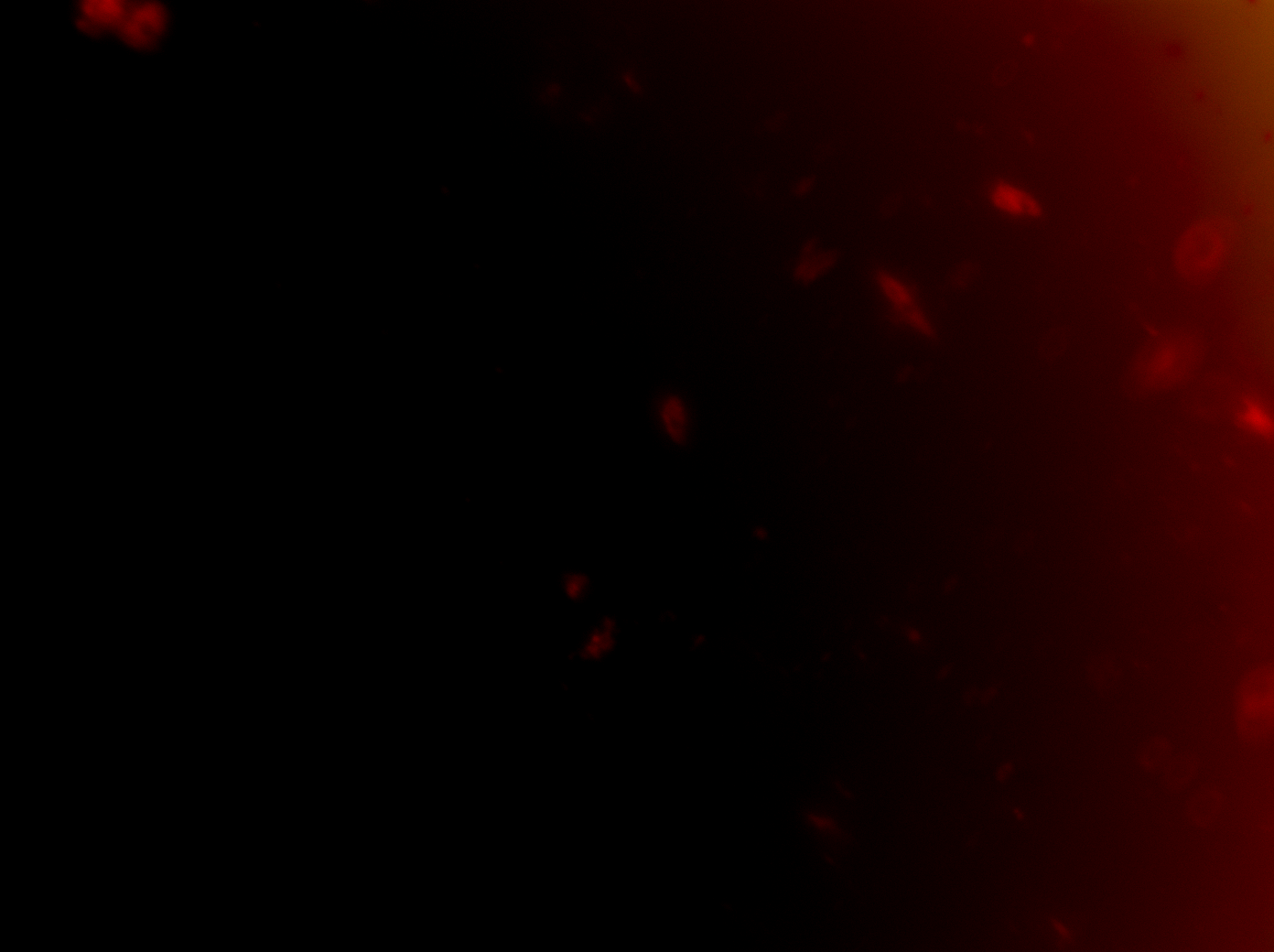

Supplement: Supplementary file 4 — Source Data [file 41467_2020_15015_MOESM4_ESM.zip › Figure 6/Figure 6c-day3.tif]

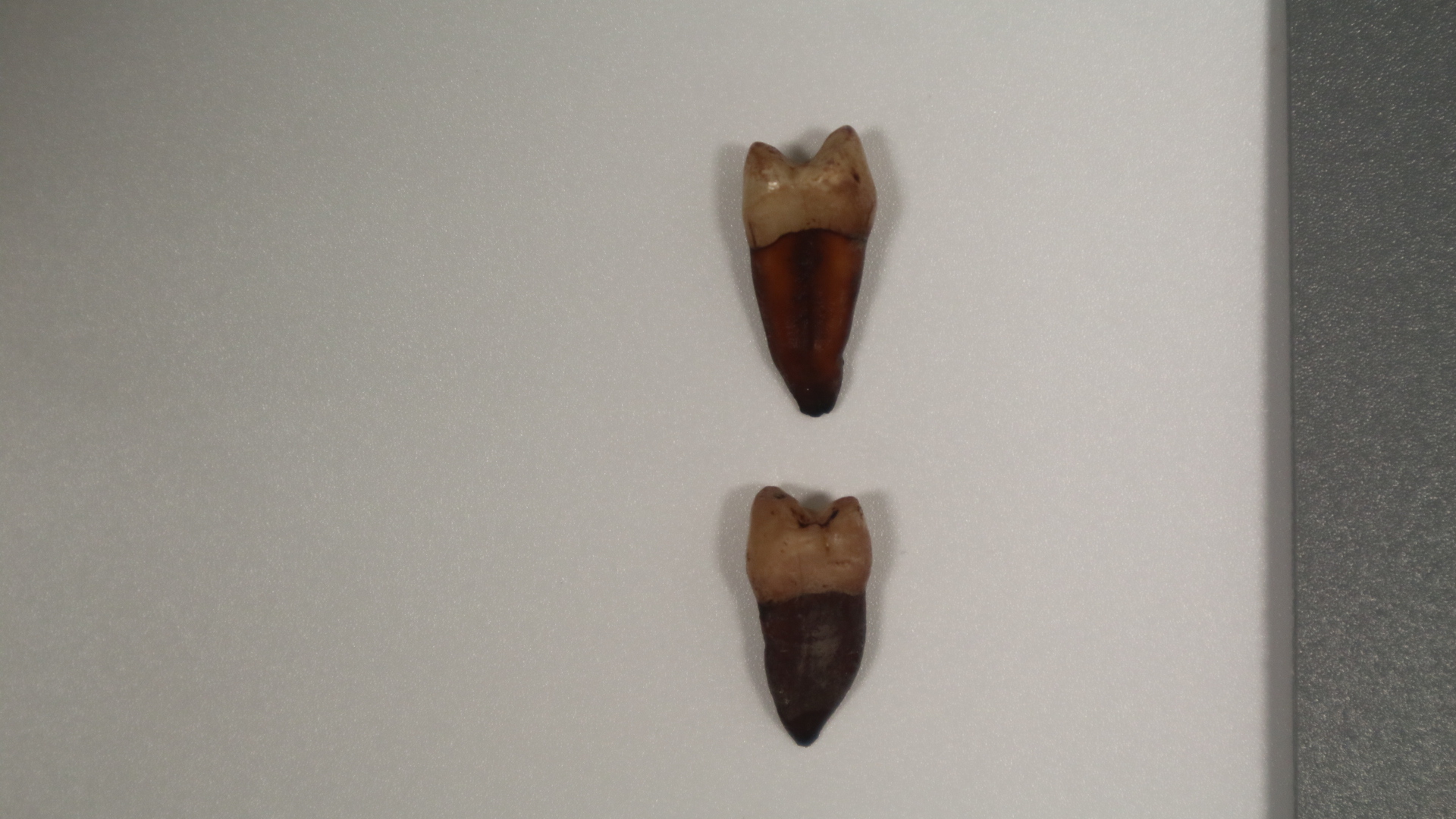

Supplement: Supplementary file 4 — Source Data [file 41467_2020_15015_MOESM4_ESM.zip › Figure4/Figure 4a 0h.JPG]

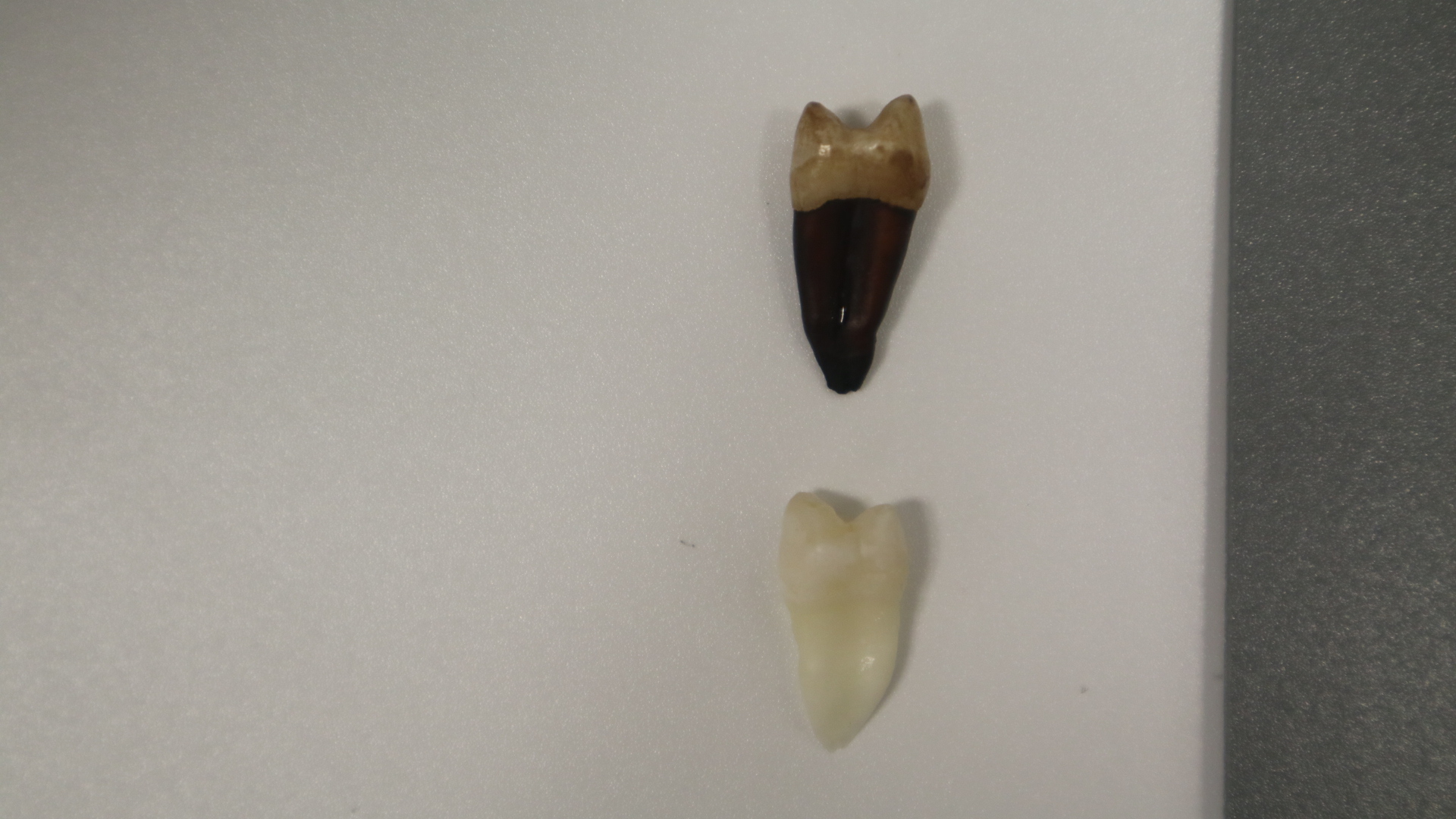

Supplement: Supplementary file 4 — Source Data [file 41467_2020_15015_MOESM4_ESM.zip › Figure4/Figure 4a 10h.JPG]

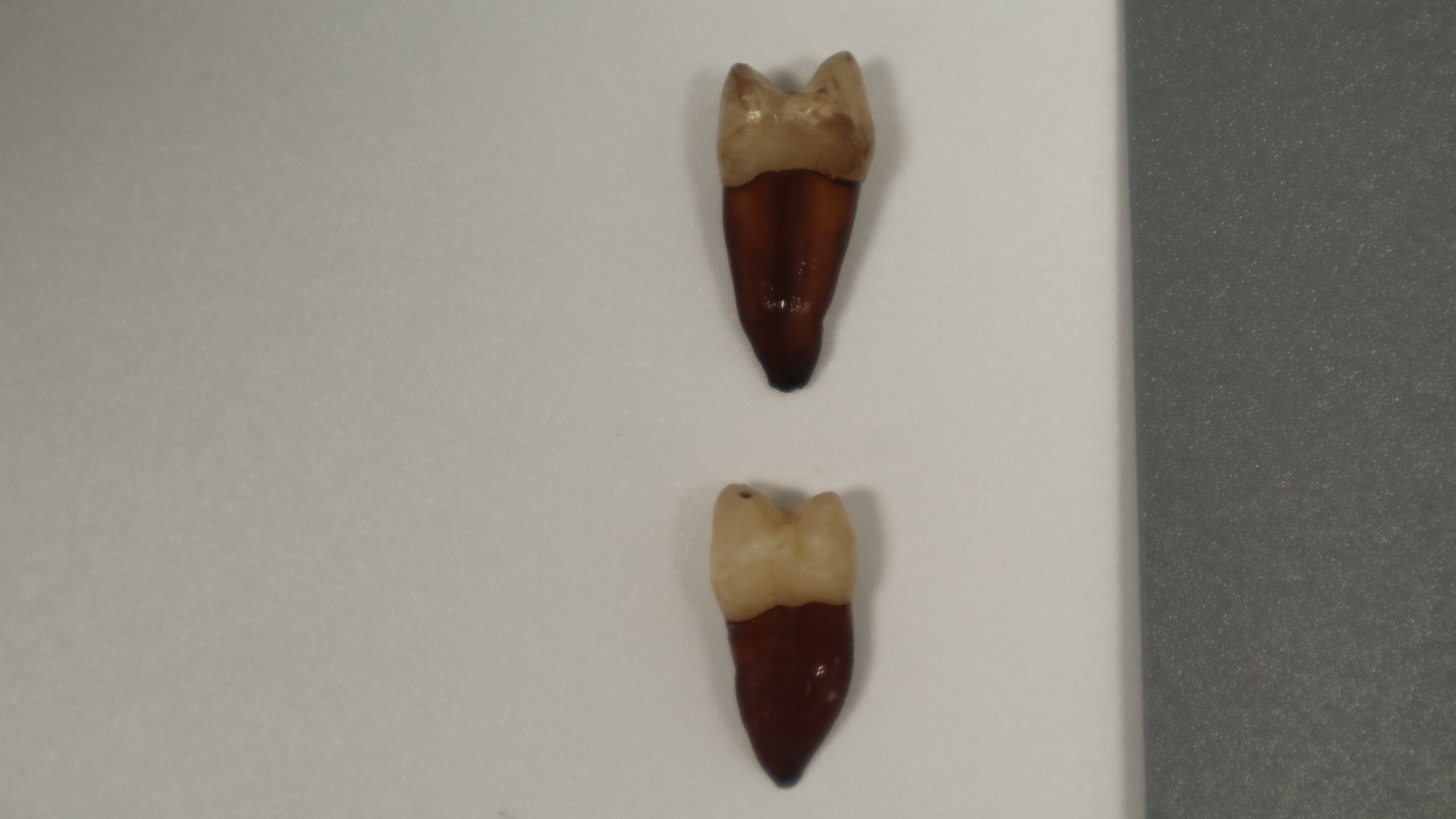

Supplement: Supplementary file 4 — Source Data [file 41467_2020_15015_MOESM4_ESM.zip › Figure4/Figure 4a 1h.JPG]

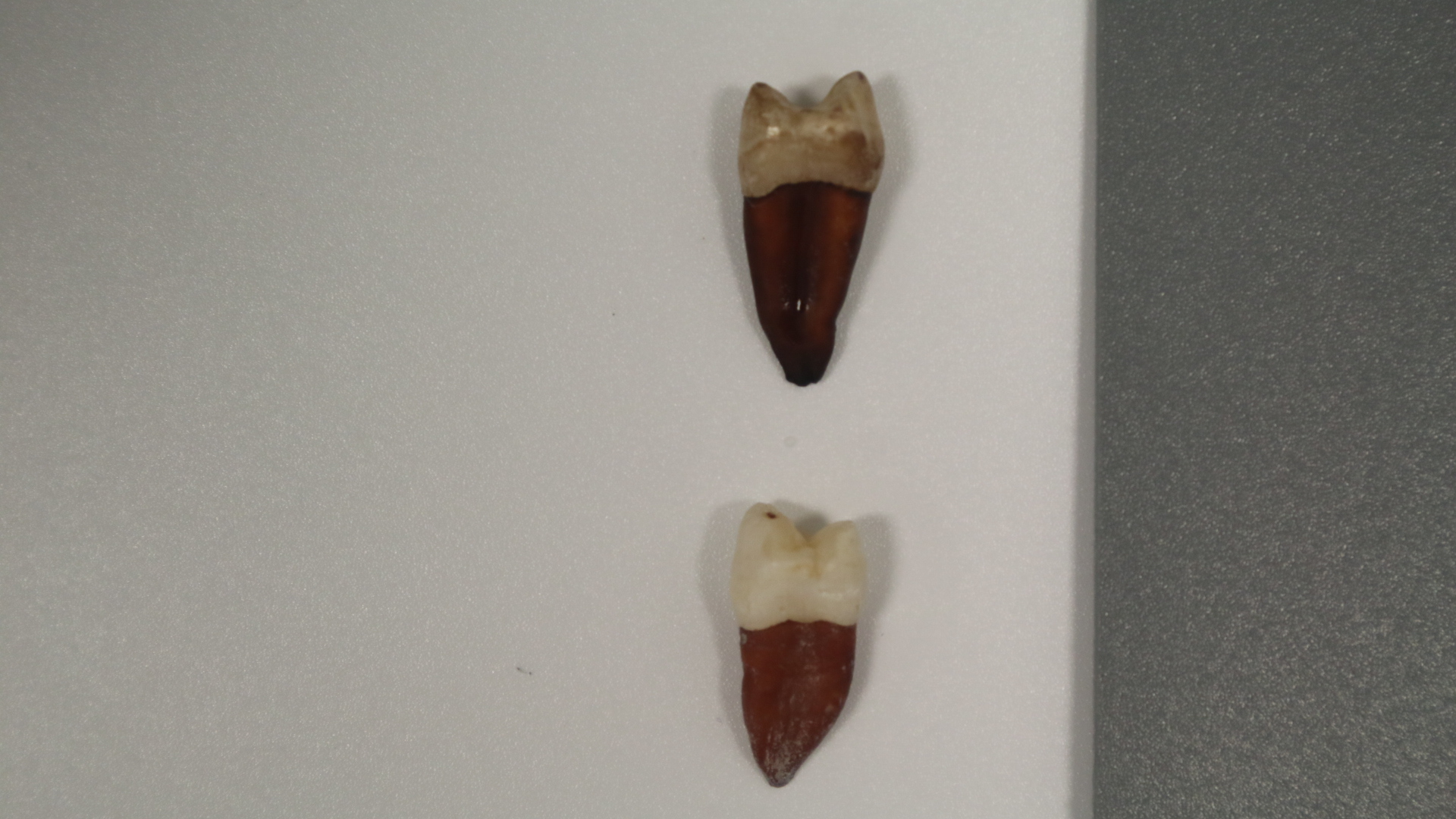

Supplement: Supplementary file 4 — Source Data [file 41467_2020_15015_MOESM4_ESM.zip › Figure4/Figure 4a 3h.JPG]

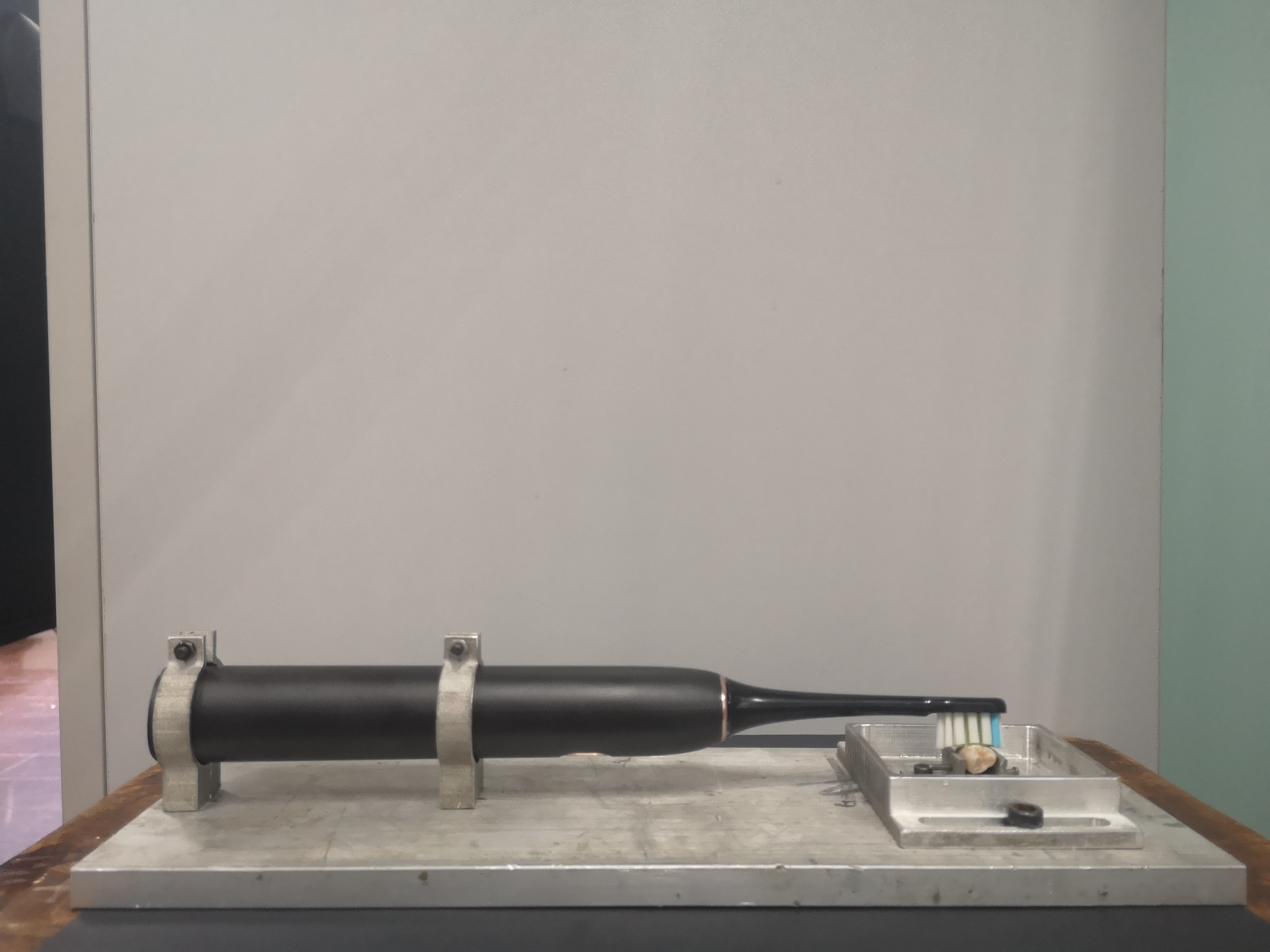

Supplement: Supplementary file 4 — Source Data [file 41467_2020_15015_MOESM4_ESM.zip › Figure4/Figure 4f.jpg]

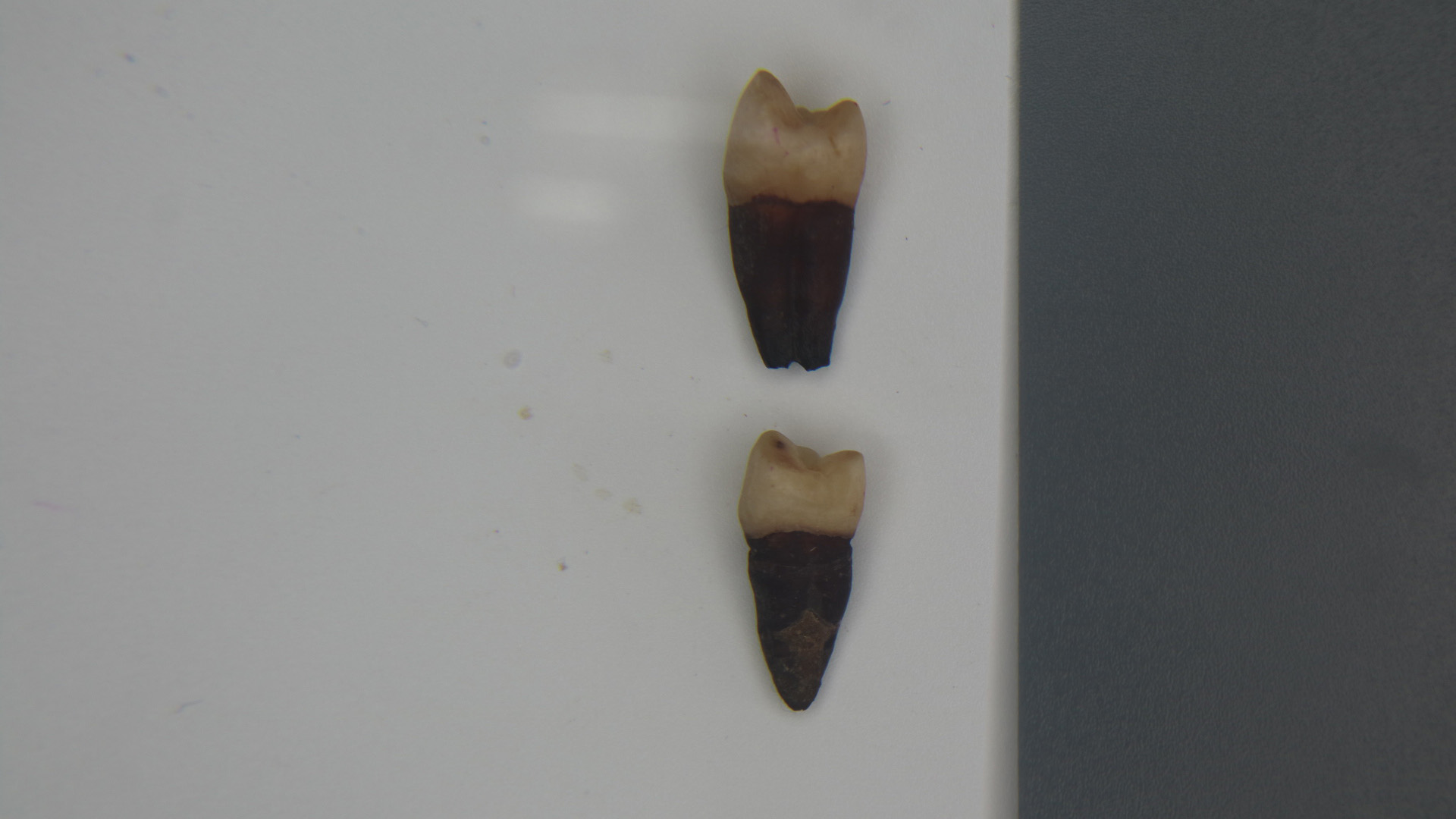

Supplement: Supplementary file 4 — Source Data [file 41467_2020_15015_MOESM4_ESM.zip › Figure4/Figure 4g 0h.JPG]

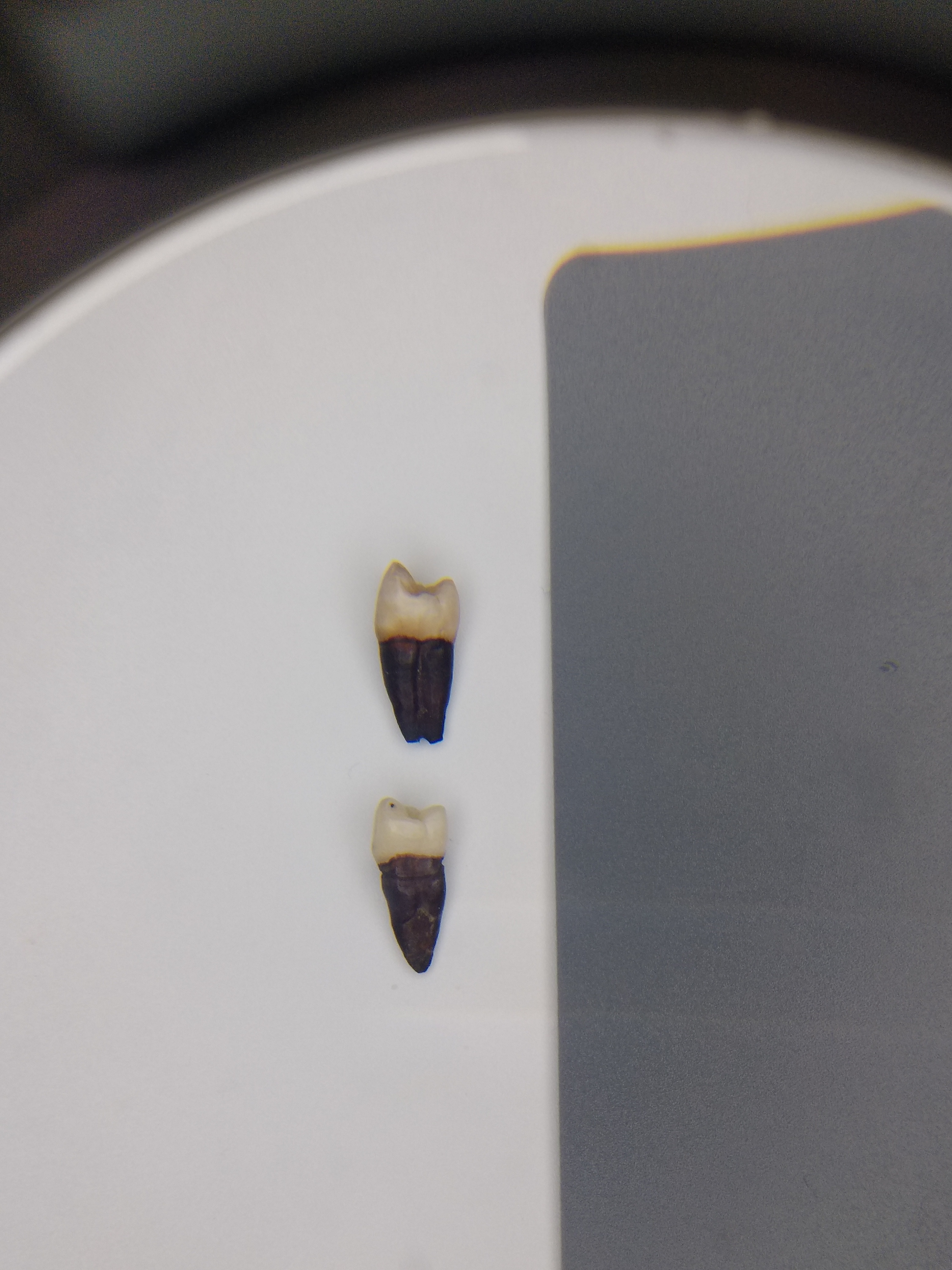

Supplement: Supplementary file 4 — Source Data [file 41467_2020_15015_MOESM4_ESM.zip › Figure4/Figure 4g 10h.jpg]
